# Light limitation and water velocity modify the impacts of simulated marine heatwaves on juvenile giant kelp

**DOI:** 10.1111/jpy.70054

**Published:** 2025-07-18

**Authors:** Imogen Bunting, Laura Bornemann Santamaría, Yun Yi Kok, Erik C. Krieger, Julia C. Mullarney, Roberta D'Archino, Christopher E. Cornwall

**Affiliations:** ^1^ School of Biological Sciences, and Coastal People Southern Skies Centre of Research Excellence Victoria University of Wellington Te Herenga Waka Wellington New Zealand; ^2^ National Institute of Water and Atmosphere Research Taihoro Nukurangi Wellington New Zealand; ^3^ Red Sea Research Centre King Abdullah University of Science and Technology Thuwal Saudi Arabia; ^4^ Coastal Marine Group, School of Science University of Waikato Hamilton New Zealand

**Keywords:** coastal darkening, kelp, light limitation, macroalgae, marine heatwaves, multi‐stressor interactions, ocean warming, water velocity

## Abstract

Coastal regions are complex habitats, where multiple natural and anthropogenic drivers can interact to affect the survival and growth of marine organisms. The giant kelp *Macrocystis pyrifera* is sensitive to increasing seawater temperatures and susceptible to marine heatwaves. Light availability and hydrodynamics can also affect the growth, morphology, and resilience of this species. In this experiment, juvenile sporophytes of *M. pyrifera* from Scorching Bay, Wellington, Aotearoa, New Zealand, a were exposed to a combination of simulated marine heatwaves at one of four different temperatures (20, 22, and 24°C compared to a 16°C control), one of two irradiance levels (shaded: 0.9 mol photons · m^−2^ · d^−1^ or ambient: 1.4 mol photons · m^−2^ · d^−1^), and one of two flow speeds (5.3 cm · s^−1^ or 6.1 cm · s^−1^) in a fully factorial design. Simulated heatwaves lasted for 21 days, with temperatures ramped by 2°C · d^−1^, followed by a 21‐day recovery phase. The heatwave treatments represented severe heatwaves in present day or hypothetical future conditions, whereas the control represented historical average summer sea temperatures in Wellington, and 21 days represented a realistic duration for heatwaves in this region. Temperature was the main driver of negative physiological impacts, with 100% of sporophytes dying within 42 days of exposure to a 24°C heatwave. Sporophytes experienced 44% mortality at 20°C and 81% mortality at 22°C, and growth rates declined significantly with increasing temperature. However, survival rates were modified by light and water velocity, with 56% of sporophytes surviving under a combination of ambient light and fast water velocity, compared with less than 50% under each of the other light‐velocity combinations. Light limitation also reduced sporophyte survival, growth rates, and effective quantum yield. Water velocity alone did not significantly affect sporophytes, but flow speeds had interactive effects with temperature and light. The findings of this experiment suggest that *M. pyrifera* at sites with optimal environmental conditions, including low sediment loads and fast tidal flows, could be more resilient to marine heatwaves, as long as temperatures do not exceed critical thresholds for survival.

**Abbreviations**
ANOVA: analysis of variance
*F*_v_′/*F*_m_′: effective quantum yield of photochemical energy conversion, measured as the ratio of variable fluorescence, *F*_v_′ to maximum fluorescence, *F*_m_′
MHW: marine heatwave
PAR: photosynthetically active radiation
RGR: relative growth rate
SSP: Shared Socioeconomic Pathway

## INTRODUCTION

Kelp forests are biodiverse marine ecosystems that are widely distributed throughout temperate coastal regions (Krumhansl et al., 2016; Mann, 1973). Kelps are ecosystem engineers that can create their own habitats by changing the physical structure of their environment (Teagle et al., 2017), modifying light availability (Arkema et al., 2009; Reed & Foster, 1984), altering waves and currents (Eckman et al., 1989; Gaylord et al., 2007), and increasing pH and dissolved oxygen concentrations in the surrounding water (Britton et al., 2016; Traiger et al., 2022). Kelp forests support high biodiversity, especially of sessile invertebrates and fish (Graham, 2004; Miller et al., 2018; Villegas et al., 2019). Hence, kelps support many commercially important fisheries (Eger et al., 2023), as well as serve as food themselves and hold cultural importance (Thurstan et al., 2018; Turner, 2001). Kelp forests can also maintain coastal stability (Løvås & Tørum, 2001) and contribute to carbon sequestration (Filbee‐Dexter & Wernberg, 2020) and nutrient cycling (Bustamante & Branch, 1996; Inglis, 1989; Vanderklift & Wernberg, 2008). Overall, kelps have been estimated to provide up to US$500 billion worth of ecosystem services globally (Eger et al., 2023). Declines in kelp abundance and health due to environmental stress can limit their contributions to ecosystem services (Blain et al., 2021; Pessarrodona et al., 2018) and greatly reduce the biomass and diversity of the biotic communities they support (Arafeh‐Dalmau et al., 2019; Johnson et al., 2011; O'Connor & Anderson, 2010).

Climate change is considered to be among the most significant stressors affecting kelps (Wernberg et al., 2019, 2023; Smale, 2020; see also literature cited within). Ocean warming has driven range shifts and declines in the abundance of numerous macroalgal species (Smale, 2020; Straub et al., 2016; Wernberg et al., 2011), as well as a reduction in the global extent of kelp forest canopies (Krumhansl et al., 2016). Marine heatwaves (MHWs), as defined by Hobday et al. (2016), are a particularly concerning aspect of climate change for kelp forest communities. Marine heatwaves are typically driven by interactions between local oceanographic and meteorological conditions and the overall warming trend linked to greenhouse gas emissions (de Burgh‐Day et al., 2022; Kerry et al., 2022; Salinger et al., 2019). These heatwaves have triggered kelp canopy collapses (Arafeh‐Dalmau et al., 2019; McPherson et al., 2021; Tolimieri et al., 2023) and regime shifts from kelp forests to turf algae beds (Wernberg et al., 2016), as well as local extinctions of cold‐temperate macroalgae such as *Scytothalia dorycarpa* (Smale & Wernberg, 2013) and *Durvillaea* spp. (Thomsen et al., 2019). Kelp forests may be more vulnerable to ocean warming and MHWs than any other temperate marine ecosystems (Cooley et al., 2023; Wernberg et al., 2023), as kelp sporophytes experience high mortality and rapid blade erosion in response to thermal stress (Fales et al., 2023; James et al., 2024).

In order to accurately predict the impacts of climate change on kelps at regional scales, it is important to understand how global changes in water temperature could interact with local‐scale drivers. In Aotearoa New Zealand, where this study was conducted, land‐use intensification has driven large increases in sediment and nutrient runoff into coastal habitats (Schiel & Howard‐Williams, 2016), which could strongly impact kelp forests. Sedimentation has had direct negative impacts on numerous species of kelp, including reduced spore settlement (Devinny & Volse, 1978; Muth et al., 2017; Phelps et al., 2024) and development (Picard et al., 2022), burial of spores and recruits (Deiman et al., 2012; Devinny & Volse, 1978), and damage from scouring (Devinny & Volse, 1978). Moreover, sediment pollution can reduce light availability in nearshore marine habitats (up to 12 m deep) by more than 50% (Blain & Shears, 2019; Desmond et al., 2015; Tait, 2019). This process of “coastal darkening” (Cornwall et al., 2023, p. 1) has been linked to declines of up to 95% in macroalgal growth rates in Aotearoa New Zealand (Blain et al., 2021; Desmond et al., 2015). Additionally, coastal darkening could facilitate regime shifts from kelp forests to habitats dominated by less productive macroalgae, such as the fucoid *Carpophyllum maschalocarpum*, which has lower light requirements (Blain & Shears, 2020). However, sediment may also protect kelp blades from harmful levels of ultraviolet radiation (Roleda et al., 2008). In laboratory studies, light limitation has been shown to greatly reduce the resilience of kelp to simulated MHWs (Bass et al., 2023; Wernberg & Straub, 2024).

Hydrodynamics can interact with other drivers on macroalgae, including temperature and light availability, in varied ways. Horizontal transport by currents and vertical mixing both affect dispersal and settlement of kelp spores, thus modulating the distribution of kelp forests and exposure of kelps to local stressors (Gaylord et al., 2012). On Vancouver Island, Canada, wave‐exposed sites have served as refugia for kelp diversity, as less heat‐tolerant species have declined in abundance due to warming at more sheltered sites (Starko et al., 2019). Heatwave‐driven losses of *Nereocystis luetkana* canopy in the Salish Sea have been most severe in areas with slow current velocities; however, this loss may be a result of increased grazing pressure in these areas rather than a direct interaction between temperature and water velocity (Berry et al., 2021). At sites where wave exposure is high enough to damage kelp blades, elevated sea surface temperatures may delay canopy recovery after storm‐driven losses (Cavanaugh et al., 2011).

The giant kelp *Macrocystis pyrifera* is one of the world's fastest‐growing and most productive macroalgae (Schiel & Foster, 2015), but it is extremely heat‐sensitive, with declines in canopy cover in many regions linked to rising sea surface temperatures (Bell et al., 2020; Butler et al., 2020) and MHWs (Arafeh‐Dalmau et al., 2019; Tait et al., 2021; Tolimieri et al., 2023). *Macrocystis pyrifera* abundance is predicted to decline worldwide under all Shared Socioeconomic Pathway (SSP) emissions scenarios (Gonzalez‐Aragon et al., 2024), and the species could become locally extinct in Australia under SSP3‐6.0 or higher (Martínez et al., 2018). Context‐dependent factors such as light availability and wave exposure can affect the responses of giant kelp to MHWs. *Macrocystis pyrifera* stands have shown critical reductions in resilience at irradiance levels below 1 mol of photosynthetically active radiation (PAR) per squared meter per day (Tait, 2019), and sediment pollution is thought to be linked to losses of *M. pyrifera* in coastal regions (Glover, 2020). Light limitation has greatly reduced the recovery rates of microscopic *M. pyrifera* sporophytes after simulated El Niño events in the laboratory (Ladah & Zertuche‐González, 2007) and can exacerbate the impacts of extreme temperatures on survival and photobiology in macroscopic sporophytes (Mabin et al., 2019). Wave exposure and abrasion driven by fast water velocity can modify recruitment dynamics and reduce survival of microscopic stages and juveniles in *M. pyrifera* (Beckley & Edwards, 2021; Graham et al., 1997). Wave exposure has also affected the morphology of *M. pyrifera* blades, which have tended to be narrower at wave‐exposed sites, perhaps because broad blades are vulnerable to damage caused by drag under high water velocity (Hurd et al., 1996; Leal et al., 2021). Adult *M. pyrifera* can grow more rapidly at more exposed sites, likely due to higher nutrient delivery rates (Hepburn et al., 2007; Stephens & Hepburn, 2014); in the laboratory, increasing water velocity by 4 cm · s^−1^ led to a 300% increase in photosynthetic output (Wheeler, 1980). Increased delivery of macro and micronutrients (Paine et al., 2023), as well as dissolved inorganic carbon, could all, thus, ameliorate some of the negative effects of MHWs on *M. pyrifera* sporophytes, whereas abrasion and drag could place additional stress on smaller sporophytes and microscopic stages. However, there has been little research into the interactions between differences in water velocity and light availability and their combined impacts on responses to MHWs.

This study aimed to quantify the potential impacts of moderate to extreme marine heatwave scenarios on *Macrocystis pyrifera* sporophytes from the warm edge of their range in New Zealand and to explore how these impacts might be modified by coastal darkening and hydrodynamics. Due to the difficulty of simulating differences in wave exposure in a laboratory setting, water velocity was used as a proxy for differences in the delivery rates of dissolved substances that could modify responses to MHWs. Based on the findings of previous studies of local *M. pyrifera* populations (Bunting et al., 2024; Le et al., 2024), it was hypothesized that kelp growth rates would negatively correlate with heatwave temperature. It was also hypothesized that sporophyte survival rates and effective quantum yield values would be adversely affected at temperatures above 20°C. Additionally, it was hypothesized that kelp survival and growth would be negatively affected by light limitation and positively affected by increased water velocity.

## MATERIALS AND METHODS

### Study site, spore collection, and culture

*Macrocystis pyrifera* sori were collected by snorkeling at low tide, at depths of 1–3 m, at Scorching Bay (41.30° S, 174.84° E) in Wellington Harbour in the spring of 2022. Approximately 50 sori were collected in total from 10 individual sporophytes spanning the depth gradient. Wellington is near the northern limit of the distribution of *M. pyrifera* in New Zealand (Hay, 1990; GBIF.org, 2025; Figure S1). The Wellington region has experienced numerous marine heatwaves in recent decades, with temperature anomalies of up to 4°C and durations ranging from 5 to over 100 days (see Bunting et al., 2024; Figures [Link], [Link]). Mean daily doses of photosynthetically active radiation (PAR) in shallow waters near Scorching Bay are ~2.2 mol photons · m^−2^ underneath the kelp canopy and 4.0 mol photons · m^−2^ at open sites (O. Peleg & A. Northmore, unpublished data). Mean flow speeds between 0.5 and 5.1 cm · s^−1^ and maximum speeds of up to 48.8 cm · s^−1^ have been recorded in the Wellington Harbour entrance, near the collection site, while faster flows of up to 69 cm · s^−1^ occur on the more exposed south coast (Carter & Lewis, 1995, and literature cited within).

*Macrocystis pyrifera* sori were transferred to the National Institute of Water and Atmosphere Research (NIWA), Taihoro Nukurangi. Spore release was stimulated through immersion in *F*/2 nutrient‐enriched filtered seawater (Guillard, 1975; AusAqua, Wallaroo, South Australia) as described in Bunting et al. (2024), and spores from all sori were mixed together and settled onto sheets of 1‐mm plastic mesh. Sporophytes were cultured on the mesh sheets in tanks of UV‐filtered seawater in a temperature‐controlled room set to 16°C. Tanks were illuminated by 4Seasons 100 W Quantum PAR LED light panels (4Seasons, Auckland, New Zealand) that provided PAR at ~ 30 μmol photons · m^−2^ · s^−1^ during a 12:12 h light:dark photoperiod. Tank temperatures were monitored as described in Bunting et al. (2024). The sporophytes used in this experiment were removed from the mesh sheets, and their holdfasts were wrapped in separate pieces of mesh. Plastic clothes pegs, with sinkers attached, were attached to these mesh parcels to keep the sporophytes fully submerged. The sporophytes were left for several weeks to reattach to the mesh before they were transferred, submerged in water, to the experimental tanks at Wellington University Coastal Ecology Laboratory on 8 February 2023. The sporophytes were ~4 months old when the experimental period commenced.

### Experimental setup

Our experimental setup was adapted from Bunting et al. (2024). The setup consisted of eight 70‐L water baths, each connected to a separate header tank and containing four 4‐L experimental tanks. Seawater was pumped continuously into the header tanks, then flowed through the experimental tanks and water baths at a rate of ~300 mL · min^−1^. Tanks were cleaned weekly to remove epiphytic algae. The total duration of the experiment was 63 days.

Water temperature was controlled by 300 W submersible heaters (EHEIM, Deizisau, Germany) and Hailea 300A 1/4HP external chillers (Hailea, Guangdong, China), connected to an Apex Classic programmable control unit (Neptune Systems, Morgan Hill, California, United States). We placed Apex temperature probes (Neptune Systems, Morgan Hill, California, United States), connected to the control unit, in one tank within each water bath. We calibrated these probes weekly against a reference thermometer (FisherBrand, Waltham, Massachusetts, United States). Heaters or chillers would be automatically switched on if the temperature in the tanks deviated from the desired temperature by 0.1°C. The Apex control unit was also connected to eight Apex pH electrodes (Neptune Systems, Morgan Hill, California, United States), which we placed in the header tanks to monitor their pH levels. We calibrated the pH probes against Orion NBS pH buffers (ThermoFisher Scientific, Waltham, Massachusetts, United States) prior to the experiment.

The tanks were illuminated by customized Zeus 70 LED panels (Ledzeal, Shenzhen, China), which emitted mostly blue and green light, on a 12:12 h light:dark cycle. Light intensity increased steadily during the first 5 h, peaked for 2 h, then decreased during the last 5 h. Water baths were surrounded by a 1‐mm mesh curtain to reduce the influence of external light sources. To simulate coastal darkening, half of the tanks in each water bath were covered with 1‐mm black plastic mesh, which reduced the daily maximum intensity of PAR within the shaded tanks from 56 to 35 μmol photons · m^−2^ · s^−1^. The total daily dose of PAR was ~1.4 mol photons · m^−2^ · d^−1^ for the fully illuminated tanks and 0.9 mol photons · m^−2^ · d^−1^ for the shaded tanks. These light treatments were chosen to simulate light availability in *Macrocystis pyrifera* habitats ~10 m deep (Desmond et al., 2015; Tait, 2019).

Water velocity was modified by equipping half of the tanks in each water bath with two 150 L · h^−1^ submersible pumps (Hailea, Guangdong, China), while the other half were fitted with just one pump. Water velocity was measured at six horizontal positions within each tank (Figure S2) and at a range of water depths, using a 10 MHz Nortek Vectrino Profiler (Nortek, Rud, Norway). Further details on measurements are given in Appendix S1. When the flow speeds for all six positions were averaged, mean flow speeds peaked at ~3 mm above the bottom of the tank, with maximum flow speeds of 6.1 cm · s^−1^ in the one‐pump treatment and 8.4 cm · s^−1^ in the two‐pump treatment with no kelp added (Figures [Link], [Link]). The variation in flow speed with depth meant that a shearing effect was observable in the two‐pump treatment (Figures S4 and S5). The mean flow speeds across all positions and depths were 5.3 cm · s^−1^ in the one‐pump treatment and 6.1 cm · s^−1^ in the two‐pump treatment.

We allocated two sporophytes haphazardly to each experimental tank and scrubbed them gently with a toothbrush once a week to remove epiphytic algae. The average sporophyte length at the beginning of the experiment was 41 ± 16 mm (mean ± standard error). Sporophytes were kept in the experimental tanks for 21 days before the simulated heatwaves commenced. The temperature in the tanks was kept between 16 and 17°C during this period to simulate typical summer sea surface temperatures throughout the Wellington region (MetOcean Solutions, 2024). Separate light and water velocity treatments were applied from the first day of the acclimation phase. This approach meant that the individual effects of light, water velocity, and temperature could be separated more easily, and allowed the sporophytes to acclimate fully to laboratory conditions before heat stress was applied.

**FIGURE 1:**
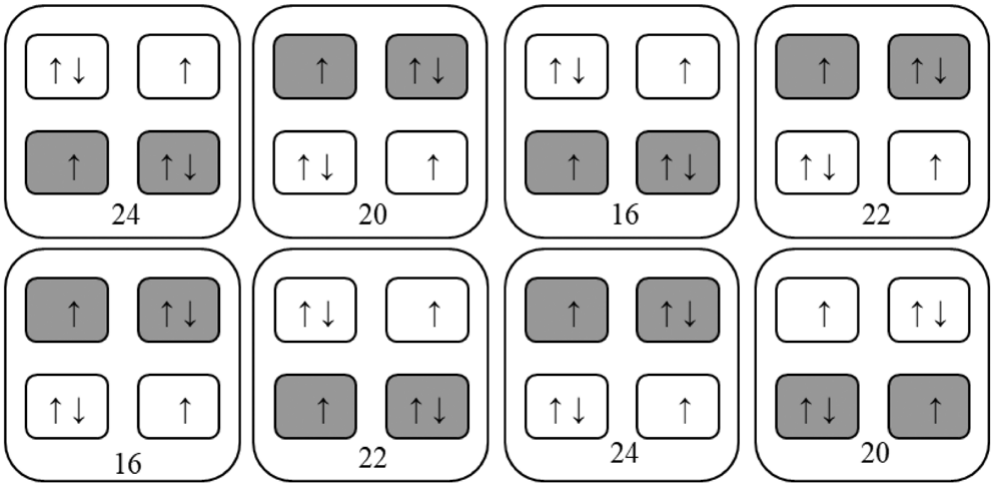
Layout of water baths and experimental tanks used in this experiment. Numbers represent temperature treatments. Arrows signify pumps. Tanks shaded in gray signify light‐limited treatments.

Four temperature treatments were used: three heatwave treatments at 20, 22, and 24°C and a control treatment of 16°C. Heatwaves ran for 21 days. At the beginning and end of the heatwave period, the temperatures were raised and lowered, respectively, by increments of 2°C per day to reduce acute thermal shock, following Sánchez‐Barredo et al. (2020) and Umanzor et al. (2021). Each heatwave treatment was applied to two water baths; the remaining two water baths were kept between 16 and 17°C throughout the experimental period to act as controls. After the heatwaves, all water baths were returned to 16°C for 21 days to observe whether the sporophytes recovered from the heatwave. The experimental design was fully factorial, with two independent replicates of each temperature × light × water velocity combination, and two sporophytes in each replicate tank, giving a total of four biological replicates. Light, water velocity, and temperature treatments were interspersed systematically (Hurlbert, 1984) to minimize any nontreatment effects (Figure 1), and water bath was used as a random factor in statistical analysis. The experimental period lasted for 63 days in total: a 21‐day acclimation phase, a 21‐day simulated heatwave phase, and a 21‐day recovery phase.

We measured temperature, pH, and dissolved oxygen concentration weekly in the experimental tanks, water baths, and header tanks for consistency. Temperature was measured using a traceable thermometer (FisherBrand, Waltham, Massachusetts, United States). pH was measured potentiometrically using an IntelliCAL PHC101 glass electrode (Hach New Zealand, Auckland, New Zealand) that was calibrated weekly against artificial seawater with Tris buffer added (Dickson et al., 2007). We used the R package seacarb (Gattuso et al., 2021) to convert the pH readings from millivolts to the total scale. Dissolved oxygen was measured with an IntelliCAL LDO101 probe (Hach New Zealand, Auckland, New Zealand), which was calibrated weekly against 0% and 100% standards. We also collected water samples for nitrogen analysis during the first week of the experimental period and the final week of each 3‐week experimental phase. We took samples from each header tank and from one experimental tank within each water bath, using methods adapted from Pritchard et al. (2015). Samples were always taken between 11:00 a.m. and 1:30 p.m. to minimize any impacts of daily fluctuations in nitrogen availability on the measured values. Sample collection, storage, and analysis techniques have been described in detail in Bunting et al. (2024). The ambient nitrogen concentration in the seawater supply was sufficient to allow growth in kelp sporophytes.

### Kelp performance

#### Mortality

Mortality rates were recorded at the end of each of the three 21‐day experimental phases. Sporophytes were marked as dead if their blades eroded completely or detached from the holdfast or if holdfasts detached from the mesh. We excluded dead sporophytes from chlorophyll fluorescence measurements, but holdfasts were weighed if they remained attached to the mesh. Some bleaching of blade tissue was observed, but bleached sporophytes were not recorded as dead unless they eroded fully.

#### Growth

We took wet weight measurements on the first day of each experimental phase, and the day after the end of the recovery period, using a set of waterproof scales (A&D Company Limited, Tokyo, Japan) with a precision of ±0.005 g. We gently blotted sporophytes dry and removed pegs and sinkers before weighing them. We also measured a reference weight each time to confirm the accuracy of the scales. Relative growth rates (RGR; Kain & Jones, 1976) were calculated for each experimental phase according to the following equation:

$$\mathrm{RGR}=\frac{\ln\left(m_{\text{final}}\right)-\ln\left(m_{\text{initial}}\right)}{t}\times 100$$

Where *m*_initial_ and *m*_final_ are the wet weight, in grams, of the sporophyte at the beginning and end of each experimental phase, and *t* is the length of time, in days, between measurements.

#### Chlorophyll fluorescence

We measured the effective quantum yield of photochemical energy conversion (the ratio of variable fluorescence, *F*_v_′, to maximum fluorescence, *F*_m_′, in the light‐adapted state) of each sporophyte on the last day of each experimental phase, using a Diving‐PAM blue light fluorometer to produce a saturating light pulse (Walz, Effeltrich, Germany). The Diving‐PAM light source was held near the base of the blade to increase consistency and ensure that the youngest tissue was assessed. We optimized the intensity (5500 μmol photons · m^−2^ · s^−1^) and duration (0.6 s) of the saturating pulse to ensure accurate measurement of *F*_v_′/*F*_m_′, as confirmed by the measurement of an n‐shaped progression in chlorophyll fluorescence during the saturating pulse.

### Statistical analysis

We used the R package survival (Therneau, 2024) to run Kaplan–Meier survival analysis (Kaplan & Meier, 1958) on the cumulative survival rates of sporophytes at the ends of the acclimation, heatwave, and recovery periods. We used the Shapiro–Wilk test (Shapiro & Wilk, 1965) to confirm that the data sets for relative growth rates and chlorophyll fluorescence were normally distributed and used the R package car (Fox et al., 2023) to carry out Levene's test for equality of variances (Levene, 1960) and confirm that variances were homogenous. We then fit linear mixed‐effects models to these data sets using the R package lme4 (Bates et al., 2023). Temperature, light intensity, and water flow, as well as their interactions, were treated as fixed effects, while water bath was treated as a random effect. Analysis of variance (ANOVA) was subsequently run on these models, and *p*‐values were generated through Type II Wald chi‐square tests, using the R package car (Fox et al., 2023). Where significant effects were determined, the R package multcomp (Hothorn et al., 2008) was used to run pairwise Tukey's tests (Tukey, 1949) on the models to assess significant differences between individual treatments and treatment combinations.

## RESULTS

### Mortality

**FIGURE 2:**
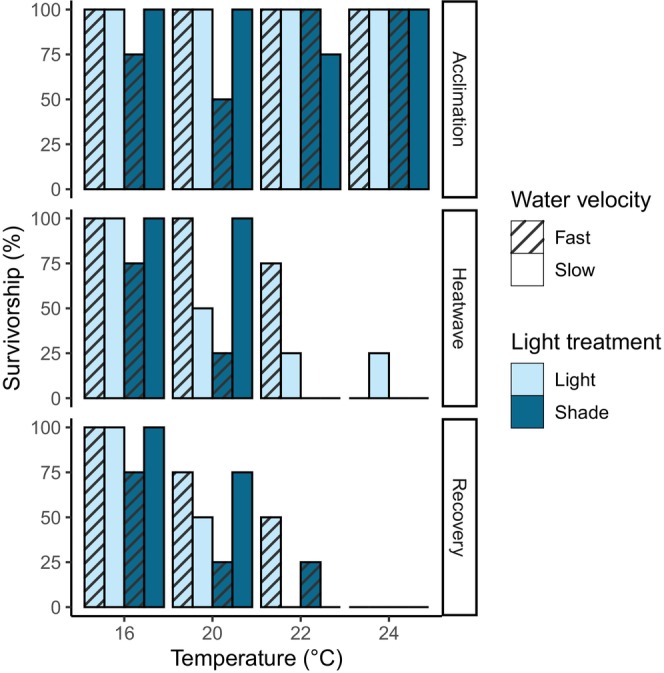
Cumulative survivorship (%) of *Macrocystis pyrifera* sporophytes in each experimental treatment at each timepoint.

Light availability had a statistically significant effect on mortality during the acclimation phase (*p* = 0.04, Kaplan–Meier analysis, χ^2^ = 4.2 on one degree of freedom; Table 1). Water velocity did not have a significant individual or interactive effect during this phase (Table 1). Four sporophytes died during the acclimation phase, all in the shaded treatments; three were in the fast water velocity treatment (Figure 2).

**TABLE 1:** *p*‐values obtained from running Kaplan–Meier survival analysis on the survival rates given in Figure 2 to assess the effects of heatwave temperature (*T*), light availability (*L*), water velocity (*V*), and their interactions on cumulative survival rates in *Macrocystis pyrifera* sporophytes during each experimental phase.

Time	*χ* ^2^	*df*	*p*
Acclimation			
*T*	2.1	3	0.60
*L*	4.2	1	**0.04**
*V*	1.0	1	0.30
*T × L*	8.4	7	0.30
*T × V*	8.4	7	0.30
*L × V*	6.3	3	0.10
*T × L × V*	21.0	15	0.10
Heatwave			
*T*	20.2	3	**<0.01**
*L*	4.2	1	**0.04**
*V*	0.2	1	0.70
*T × L*	26.8	7	**<0.01**
*T × V*	22.9	7	**<0.01**
*L × V*	8.1	3	**0.04**
*T × L × V*	37.0	15	**<0.01**
Recovery			
*T*	30.5	3	**<0.01**
*L*	2.9	1	0.09
*V*	0.0	1	1.00
*T × L*	36.3	7	**<0.01**
*T × V*	33.4	7	**<0.01**
*L × V*	5.5	3	0.10
*T × L × V*	43.5	15	**<0.01**

*Note*: Chi‐squared (χ^2^) values and degrees of freedom (*df*) are provided. Statistically significant effects (*p* < 0.05) are indicated in bold.

Temperature was the main driver of cumulative mortality by the end of the heatwave (*p* < 0.01, Kaplan–Meier analysis, χ^2^ = 20.2 on three degrees of freedom; Table 1), although light also had a significant independent effect (*p* = 0.04, Kaplan–Meier analysis, χ^2^ = 4.2 on one degree of freedom; Table 1), with survival rates being lower overall in the shaded treatments (Figure 2). Interactive effects were also observed between all three drivers and all combinations of drivers (Table 1). During the heatwave phase, 84% of all sporophytes in the 22 and 24°C treatments died; the only notable exception to this trend was the 22°C treatment with ambient light and fast water velocity, in which only one of the four sporophytes died. Some deaths also occurred at 20°C, but only in the ambient light, slow water velocity treatment (50% mortality) and the shaded, fast water velocity treatment (75% mortality; Figure 2).

Temperature was the most significant driver of cumulative mortality throughout the entire experimental period (*p* < 0.01, Kaplan–Meier analysis, χ^2^ = 30.5 on three degrees of freedom; Table 1), whereas light and water velocity did not have significant individual impacts. Interactive effects were observed between temperature and light, between temperature and water velocity, and among all three drivers (Table 1). Five sporophytes died during the recovery phase (Figure 2). One sporophyte in the 22°C treatment with shade and fast velocity was marked as dead after the heatwave but regrew some blade tissue during the recovery period and was therefore recorded as alive at the end of the experiment. No deaths occurred in the 16°C treatments during the heatwave or recovery phase. Overall, a combination of ambient light and faster water velocity seemed to be advantageous, with 56% of all sporophytes in this treatment surviving to the end of the recovery phase, whereas less than 50% of sporophytes survived under the other light‐velocity combinations, and just 31% survived under a combination of light limitation and fast water velocity (Figure 2). Due to the complete mortality in the 24°C treatments and the very low mortality in the control treatments, the impacts of shade and water velocity on survival rates were only observable at 20 and 22°C; hence, these drivers had significant interactive effects with temperature, even though their individual impacts over the entire experimental period were not significant.

### Growth

**FIGURE 3:**
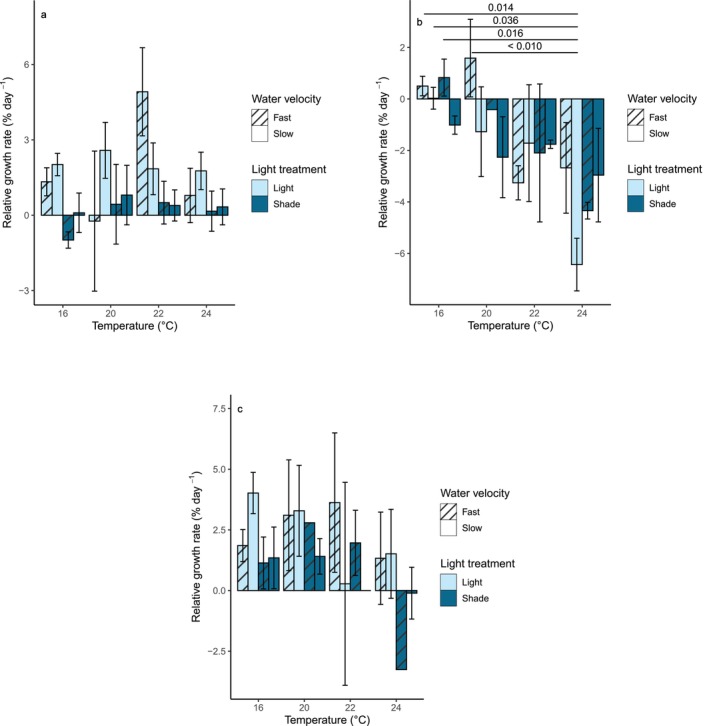
Mean relative growth rates (measured as wet weight), with standard error, of *Macrocystis pyrifera* sporophytes in each experimental treatment during (a) the acclimation phase, (b) the heatwave, and (c) the recovery phase. Significant differences between treatments are indicated by connecting lines, with *p*‐values provided.

Light availability had a statistically significant effect on RGRs (*p* < 0.01, ANOVA, χ^2^ = 7.2 on 1 degree of freedom; Table 2) during the acclimation phase; mean RGRs were much higher in the ambient light treatment than the shaded treatment (Figure 3a; Table S1). Water velocity had no significant impact on RGR, and did not modify the impact of light availability (Table 2; Figure 3a). There were no significant differences between individual treatment combinations (Table 3a).

**TABLE 2:** *p*‐values obtained from fitting linear mixed‐effects models to the data collected during each phase of the heatwave experiment to assess the effects of heatwave temperature (*T*), light availability (*L*), water velocity (*V*), and their interactions on relative growth rates (RGR) in *Macrocystis pyrifera* sporophytes.

Time	Group *df*	χ^2^	Residual *df*	*p*
Acclimation				
*T*	52.0	0.349	1	0.554
*L*	52.0	7.242	1	**0.007**
*V*	52.0	0.368	1	0.544
*T × L*	52.0	0.058	1	0.810
*T × V*	52.0	0.485	1	0.486
*L × V*	52.0	0.004	1	0.949
*T × L × V*	52.0	0.007	1	0.932
Heatwave				
*T*	36.0	19.785	1	**<0.001**
*L*	36.0	0.267	1	0.605
*V*	36.0	2.737	1	0.098
*T × L*	36.0	0.480	1	0.489
*T × V*	36.0	0.148	1	0.700
*L × V*	36.0	0.380	1	0.538
*T × L × V*	36.0	1.691	1	0.193
Recovery				
*T*	32.8	0.176	1	0.675
*L*	26.8	2.946	1	0.086
*V*	26.7	0.018	1	0.893
*T × L*	27.2	0.314	1	0.575
*T × V*	27.1	0.829	1	0.363
*L × V*	26.8	0.007	1	0.933
*T × L × V*	27.1	0.795	1	0.373

*Note*: Water bath was treated as a random effect. Chi‐squared (χ^2^) values and degrees of freedom are provided. Statistically significant effects (*p* < 0.05) are indicated in bold.

**TABLE 3:** *p*‐values obtained from pairwise Tukey's tests assessing differences in relative growth rates (RGR) of *Macrocystis pyrifera* sporophytes between experimental treatments during (a) the acclimation phase, (b) the heatwave, and (c) the recovery phase.

(a)																		
Temperature (°C)			16				20				22				24			
Light treatment			Light		Shade		Light		Shade		Light		Shade		Light		Shade	
Water velocity			Fast	Slow	Fast	Slow	Fast	Slow	Fast	Slow	Fast	Slow	Fast	Slow	Fast	Slow	Fast	Slow
16	Light	Fast																
		Slow	1.000															
	Shade	Fast	0.996	0.951														
		Slow	1.000	0.999	1.000													
20	Light	Fast	1.000	0.993	1.000	1.000												
		Slow	1.000	1.000	0.823	0.982	0.945											
	Shade	Fast	1.000	1.000	1.000	1.000	1.000	1.000										
		Slow	1.000	1.000	1.000	1.000	1.000	1.000	1.000									
22	Light	Fast	0.715	0.930	0.070	0.202	0.123	0.990	0.683	0.474								
		Slow	1.000	1.000	0.970	1.000	0.997	1.000	1.000	1.000	0.893							
	Shade	Fast	1.000	1.000	1.000	1.000	1.000	0.997	1.000	1.000	0.343	1.000						
		Slow	1.000	1.000	1.000	1.000	1.000	0.998	1.000	1.000	0.439	1.000	1.000					
24	Light	Fast	1.000	1.000	1.000	1.000	1.000	1.000	1.000	1.000	0.467	1.000	1.000	1.000				
		Slow	1.000	1.000	0.977	1.000	0.998	1.000	1.000	1.000	0.869	1.000	1.000	1.000	1.000			
	Shade	Fast	1.000	0.999	1.000	1.000	1.000	0.986	1.000	1.000	0.222	1.000	1.000	1.000	1.000	1.000		
		Slow	1.000	1.000	1.000	1.000	1.000	0.993	1.000	1.000	0.279	1.000	1.000	1.000	1.000	1.000	1.000	

(b)																		
Temperature (°C)			16				20				22				24			
Light treatment			Light		Shade		Light		Shade		Light		Shade		Light		Shade	
Water velocity			Fast	Slow	Fast	Slow	Fast	Slow	Fast	Slow	Water velocity	Fast	Slow	Fast	Slow	Fast	Slow	Fast
16	Light	Fast																
		Slow	1.000															
	Shade	Fast	1.000	1.000														
		Slow	1.000	1.000	0.999													
20	Light	Fast	1.000	1.000	1.000	0.935												
		Slow	1.000	1.000	0.999	1.000	0.974											
	Shade	Fast	1.000	1.000	1.000	1.000	1.000	1.000										
		Slow	0.896	0.978	0.868	1.000	0.413	1.000	1.000									
22	Light	Fast	0.598	0.803	0.568	0.992	0.166	1.000	0.999	1.000								
		Slow	0.998	1.000	0.995	1.000	0.914	1.000	1.000	1.000	1.000							
	Shade	Fast	0.990	0.999	0.980	1.000	0.817	1.000	1.000	1.000	1.000	1.000						
		Slow	0.998	1.000	0.994	1.000	0.905	1.000	1.000	1.000	1.000	1.000	1.000					
24	Light	Fast	0.840	0.953	0.805	1.000	0.368	1.000	1.000	1.000	1.000	1.000	1.000	1.000				
		Slow	**0.014**	**0.036**	**0.016**	0.184	**< 0.010**	0.514	0.601	0.638	1.000	0.673	0.794	0.688	0.852			
	Shade	Fast	0.366	0.556	0.342	0.910	0.088	0.987	0.980	0.999	1.000	0.998	1.000	0.998	1.000	1.000		
		Slow	0.879	0.962	0.844	1.000	0.482	1.000	1.000	1.000	1.000	1.000	1.000	1.000	1.000	0.960	1.000	

Temperature (°C)	16	20	22
20	0.918		
22	**0.048**	0.246	
24	**< 0.001**	**0.002**	0.386

(c)																		
Temperature (°C)			16				20				22				24			
Light treatment			Light		Shade		Light		Shade		Light		Shade		Light		Shade	
Water velocity			Fast	Slow	Fast	Slow	Fast	Slow	Fast	Slow	Fast	Slow	Fast	Slow	Fast	Slow	Fast	Slow
16	Light	Fast																
		Slow	0.994															
	Shade	Fast	1.000	0.930														
		Slow	1.000	0.958	1.000													
20	Light	Fast	1.000	1.000	1.000	1.000												
		Slow	1.000	1.000	1.000	1.000	1.000											
	Shade	Fast	1.000	1.000	1.000	1.000	1.000	1.000										
		Slow	1.000	1.000	1.000	1.000	0.997	1.000	1.000									
22	Light	Fast	1.000	1.000	1.000	1.000	1.000	1.000	1.000	1.000								
		Slow	1.000	1.000	1.000	1.000	1.000	1.000	1.000	1.000	1.000							
	Shade	Fast	1.000	1.000	1.000	1.000	1.000	1.000	1.000	1.000	1.000	1.000						
		Slow	1.000	1.000	1.000	1.000	1.000	1.000	1.000	1.000	1.000	1.000	1.000					
24	Light	Fast	1.000	1.000	1.000	1.000	1.000	1.000	1.000	1.000	1.000	1.000	1.000	1.000				
		Slow	1.000	1.000	1.000	1.000	1.000	1.000	1.000	1.000	1.000	1.000	1.000	1.000	1.000			
	Shade	Fast	1.000	0.967	1.000	1.000	0.985	1.000	1.000	1.000	0.997	1.000	0.983	1.000	0.994	0.999		
		Slow	1.000	1.000	1.000	1.000	1.000	1.000	1.000	1.000	1.000	1.000	1.000	1.000	1.000	1.000	0.999	

*Note*: Tukey's test results for the individual effect of temperature are included where this was statistically significant (see Table 2). Statistically significant (*p* < 0.05) differences between treatments are indicated in bold.

Temperature had a significant effect on RGR (*p* < 0.01, ANOVA, χ^2^ = 19.8 on one degree of freedom; Table 2) during the heatwave phase, whereas light and water velocity had no significant effects during this phase (Table 2). There was a consistent negative relationship between temperature and mean RGR, with a sharp decline at 24°C (Figure 3b; Table S1). Mean RGR values tended to be more positive in the ambient light tanks at 16 and 20°C (Figure 3b; Table S1), but this difference was not statistically significant and was not observed at 22 or 24°C. Relative growth rate values were also more negative, on average, in the slow velocity treatments than the fast velocity treatments (Table S1), but this was not statistically significant. Pairwise Tukey's tests determined that RGR values at 24°C were significantly lower than those at either 16 or 20°C (*p* < 0.01, Tukey's pairwise tests, *z* = −4.35 and *z* = −3.52, respectively; Table 3b), while RGR values at 22°C were also significantly lower than those in the 16°C control treatment (*p* = 0.05, Tukey's pairwise test, *z* = −2.58; Table 3b). When comparing treatment combinations, the treatment subjected to a 24°C heatwave, ambient light, and slow water velocity had significantly lower RGR values than three of the 16°C treatment combinations (*p* < 0.04, Tukey's pairwise tests, *z* ≤ −3.51, Table 3b; Figure 3b) as well as the 20°C, ambient light, fast water velocity treatment (*p* < 0.01, Tukey's pairwise test, *z* = −4.36, Table 3b; Figure 3b).

None of the experimental drivers had any significant impacts on RGR during the recovery phase, although light had a near‐significant impact (*p* = 0.09, ANOVA, χ^2^ = 2.9 on one degree of freedom; Table 2), and there were no significant differences between treatment combinations (Table 3c). Most surviving sporophytes increased in mass during the recovery phase (Figure 3c; Table S1). The 24°C treatments were an exception to this, as half of the remaining holdfasts in these treatments continued to decline in mass. Relative growth rates were much higher, on average, in the ambient light treatments than in the shaded treatments (Table S1), but this was not statistically significant.

### Effective quantum yield

**FIGURE 4:**
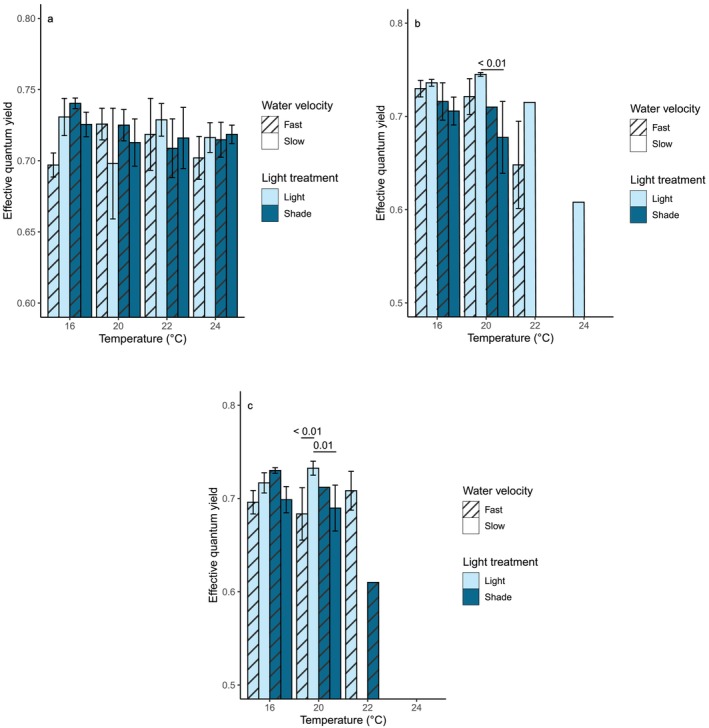
Mean effective quantum yield (*F*_v_′/*F*_m_′), with standard error, of *Macrocystis pyrifera* sporophytes in each experimental treatment during (a) the acclimation phase, (b) the heatwave, and (c) the recovery phase. Significant differences between treatments are indicated by connecting lines, with *p*‐values provided.

There was no consistent variation in *F*_v_′/*F*_m_′ between treatments during the acclimation phase (Figure 4a); the mean value across all treatments was 0.717 ± 0.004. None of the experimental stressors had any statistically significant effects on *F*_v_′/*F*_m_′ during the acclimation phase (Tables 4 and 5a). Light availability had a significant impact on *F*_v_′/*F*_m_′ during the heatwave (*p* < 0.01, ANOVA, χ^2^ = 9.8 on one degree of freedom; Table 4), with mean *F*_v_′/*F*_m_′ values being higher under ambient light than under shade (Table S2). Although water velocity did not have a significant independent effect on *F*_v_′/*F*_m_′ (*p* = 0.30, ANOVA, χ^2^ = 1.1 on one degree of freedom; Table 4), there was a significant interactive effect of light and water velocity (*p* = 0.01, ANOVA, χ^2^ = 5.9 on one degree of freedom; Table 4), as well as an interactive effect of all three drivers (*p* < 0.01, ANOVA, χ^2^ = 6.8 on one degree of freedom; Table 4). Mean *F*_v_′/*F*_m_′ values were highest under a combination of ambient light and slow water velocity and lowest under a combination of shade and slow water velocity (Table S2). During the heatwave phase, *F*_v_′/*F*_m_′ values declined steeply throughout the 22°C treatments to an average of 0.665 ± 0.037 (Table S2), while the one surviving sporophyte at 24°C had a similarly low *F*_v_′/*F*_m_′ value of 0.608. Conversely, there was little change in average *F*_v_′/*F*_m_′ values among the sporophytes kept at 16 or 20°C (Figure 4b). However, the effect of temperature on *F*_v_′/*F*_m_′ during the heatwave was not statistically significant (*p* = 0.12, ANOVA, χ^2^ = 2.4 on one degree of freedom; Table 4). The only significant difference between individual treatment combinations occurred among the 20°C treatments; sporophytes kept under a combination of ambient light and slow water velocity at 20°C had significantly higher *F*_v_′/*F*_m_′ values than the sporophytes under shade and low water velocity at this temperature (*p* < 0.01, Tukey's pairwise test, *z* = −3.98, Table 5b; Figure 4b).

**TABLE 4:** *p*‐values obtained from fitting linear mixed‐effects models to the data collected during each phase of the heatwave experiment to assess the effects of heatwave temperature (*T*), light availability (*L*), water velocity (*V*), and their interactions on effective quantum yield (*F*_v_′/*F*_m_′) on *Macrocystis pyrifera* sporophytes.

Time	Group *df*	χ^2^	Residual *df*	*p*
Acclimation				
*T*	51.7	0.302	1	0.582
*L*	45.9	0.358	1	0.550
*V*	45.8	0.138	1	0.710
*T × L*	45.9	0.575	1	0.448
*T × V*	45.8	0.006	1	0.937
*L × V*	46.0	0.375	1	0.540
*T × L × V*	46.0	0.790	1	0.374
Heatwave				
*T*	5.3	2.374	1	0.123
*L*	17.3	9.797	1	**0.002**
*V*	19.7	1.089	1	0.297
*T × L*	17.3	1.509	1	0.219
*T × V*	20.2	1.465	1	0.226
*L × V*	18.0	5.917	1	**0.014**
*T × L × V*	18.2	6.824	1	**0.009**
Recovery				
*T*	4.6	0.109	1	0.741
*L*	18.3	1.596	1	0.206
*V*	18.2	1.572	1	0.210
*T × L*	18.6	4.946	1	**0.026**
*T × V*	18.4	2.483	1	0.115
*L × V*	17.7	6.574	1	**0.010**
*T × L × V*	18.0	0.045	1	0.833

*Note*: Water bath was treated as a random effect. Chi‐squared (χ^2^) values and degrees of freedom are provided. Statistically significant effects (*p* < 0.05) are indicated in bold.

**TABLE 5:** *p*‐values obtained from pairwise Tukey's tests assessing differences in effective quantum yield (*F*_v_′/*F*_m_′) values of *Macrocystis pyrifera* sporophytes between experimental treatments during (a) the acclimation phase, (b) the heatwave, and (c) the recovery phase.

(a)																		
Temperature (°C)			16				20				22				24			
Light treatment			Light		Shade		Light		Shade		Light		Shade		Light		Shade	
Water velocity			Fast	Slow	Fast	Slow	Fast	Slow	Fast	Slow	Water velocity	Fast	Slow	Fast	Slow	Fast	Slow	Fast
16	Light	Fast																
		Slow	0.980															
	Shade	Fast	0.920	1.000														
		Slow	0.996	1.000	1.000													
20	Light	Fast	0.999	1.000	1.000	1.000												
		Slow	1.000	0.998	0.983	1.000	0.997											
	Shade	Fast	1.000	1.000	1.000	1.000	1.000	1.000										
		Slow	1.000	1.000	1.000	1.000	1.000	1.000	1.000									
22	Light	Fast	1.000	1.000	1.000	1.000	1.000	1.000	1.000	1.000								
		Slow	0.998	1.000	1.000	1.000	1.000	0.999	1.000	1.000	1.000							
	Shade	Fast	1.000	1.000	0.999	1.000	1.000	1.000	1.000	1.000	1.000	1.000						
		Slow	1.000	1.000	1.000	1.000	1.000	1.000	1.000	1.000	1.000	1.000	1.000					
24	Light	Fast	1.000	0.999	0.993	1.000	1.000	1.000	1.000	1.000	1.000	1.000	1.000	1.000				
		Slow	1.000	1.000	1.000	1.000	1.000	1.000	1.000	1.000	1.000	1.000	1.000	1.000	1.000			
	Shade	Fast	1.000	1.000	1.000	1.000	1.000	1.000	1.000	1.000	1.000	1.000	1.000	1.000	1.000	1.000		
		Slow	1.000	1.000	1.000	1.000	1.000	1.000	1.000	1.000	1.000	1.000	1.000	1.000	1.000	1.000	1.000	

(b)																		
Temperature (°C)			16				20				22				24			
Light treatment			Light		Shade		Light		Shade		Light		Shade		Light		Shade	
Water velocity			Fast	Slow	Fast	Slow	Fast	Slow	Fast	Slow	Water velocity	Fast	Slow	Fast	Slow	Fast	Slow	Fast
16	Light	Fast																
		Slow	1.000															
	Shade	Fast	1.000	0.997														
		Slow	0.984	0.923	1.000													
20	Light	Fast	1.000	1.000	1.000	1.000												
		Slow	0.992	0.997	0.965	0.918	0.276											
	Shade	Fast	1.000	1.000	1.000	0.999	0.995	0.996										
		Slow	0.996	0.991	1.000	1.000	0.546	**<0.010**	0.438									
22	Light	Fast	0.807	0.739	0.930	0.966	0.883	0.201	0.659	0.999								
		Slow	1.000	1.000	1.000	0.999	1.000	1.000	1.000	0.973	0.091							
	Shade	Fast																
		Slow																
24	Light	Fast																
		Slow	0.793	0.738	0.899	0.941	0.857	0.274	0.651	0.995	1.000	0.670						
	Shade	Fast																
		Slow																

Light treatment		Light		Shade	
Water velocity		Fast	Slow	Fast	Slow
Light	Fast				
	Slow	0.897			
Shade	Fast	0.843	0.998		
	Slow	0.889	1.000	0.999	

(c)																		
Temperature (°C)			16				20				22				24			
Light treatment			Light		Shade		Light		Shade		Light		Shade		Light		Shade	
Water velocity			Fast	Slow	Fast	Slow	Fast	Slow	Fast	Slow	Fast	Slow	Fast	Slow	Fast	Slow	Fast	Slow
16	Light	Fast																
		Slow	0.962															
	Shade	Fast	0.924	1.000														
		Slow	1.000	0.985	0.957													
20	Light	Fast	1.000	0.998	0.990	1.000												
		Slow	0.752	0.959	0.992	0.791	**<0.010**											
	Shade	Fast	0.974	0.999	1.000	0.982	0.338	0.999										
		Slow	1.000	1.000	0.997	1.000	1.000	**0.013**	0.487									
22	Light	Fast	1.000	1.000	1.000	1.000	1.000	0.844	0.989	1.000								
		Slow																
	Shade	Fast	0.910	0.666	0.587	0.985	0.975	0.111	0.362	0.950	0.417							
		Slow																

Temperature (°C)		16		20		22	
Light treatment		Light	Shade	Light	Shade	Light	Shade
16	Light						
	Shade	1.000					
20	Light	1.000	1.000				
	Shade	0.999	0.999	0.999			
22	Light	1.000	1.000	1.000	1.000		
	Shade	0.287	0.289	0.321	0.438	0.279	

Light treatment	Light		Shade	
Water velocity	Fast	Slow	Fast	Slow
Light	Fast			
	Slow	0.145		
Shade	Fast	0.894	0.726	
	Slow	1.000	0.148	0.891

*Notes*: Tukey's test results for two‐way interactions are included where these were statistically significant (see Table 4). Statistically significant (*p* < 0.05) differences between treatments are indicated in bold.

None of the three experimental drivers had significant individual effects on *F*_v_′/*F*_m_′ during the recovery phase (Table 4), but significant interactive effects were seen between temperature and light (*p* = 0.03, ANOVA, χ^2^ = 4.9 on one degree of freedom; Table 4) and light and water velocity (*p* = 0.01, ANOVA, χ^2^ = 6.6 on one degree of freedom; Table 4). *F*_v_′/*F*_m_′ values were generally low in the shaded tanks that had been exposed to a 22°C heatwave, although this was not statistically significant, and highest under a combination of ambient light and slow water velocity (Figure 4c; Table S2). Once again, significant differences were observed between the 20°C treatments; sporophytes that had been exposed to a 20°C heatwave under ambient light and slow water velocity had significantly higher *F*_v_′/*F*_m_′ values than those kept under a combination of ambient light and fast water velocity or shade and slow water velocity at that temperature (Table 5c; Figure 4c).

## DISCUSSION

We observed that increased temperature and light limitation could both independently reduce the survival and growth of young *Macrocystis pyrifera* sporophytes, while light limitation also reduced their effective quantum yield. Water velocity alone did not have a significant impact on *M. pyrifera*, but it could modify the effects of temperature and light availability. Temperature was the most critical driver at extreme values (22°C and above), while light and water velocity were also important drivers at moderate temperatures.

The impacts of thermal stress on survival and growth rates in *Macrocystis pyrifera* and other kelp species have often been more severe in light‐limited conditions (Bass et al., 2023; Mabin et al., 2019; Wernberg & Straub, 2024), as they were in this experiment. Respiration rates typically increased with temperature in algae, resulting in an increased demand for carbohydrates, which was often met by a corresponding increase in photosynthetic rates up to a maximum value (Coelho et al., 2000; Davison et al., 1991). Elevated photosynthetic rates, in turn, led to an increase in critical light requirements with increasing temperature in kelps (Staehr & Wernberg, 2009). Hence, when photosynthetic rates are constrained by light limitation, kelps may not be able to satisfy their carbohydrate demands for respiration and cell division, leading to a reduction in growth rates and increased tissue necrosis (Bass et al., 2023; Wernberg & Straub, 2024). In some regions of New Zealand, losses of *M. pyrifera* canopy cover during MHWs have been exacerbated by turbidity (Tait et al., 2021), indicating that both these stressors may threaten the long‐term persistence of *M. pyrifera* near the warm edge of its range (Cornwall et al., 2023).

Survival rates were highest under a combination of ambient light and fast water velocity across all temperature treatments; the shade‐fast velocity treatments had the lowest survival rates, as well as generally low *F*_v_′/*F*_m_′ values during and after heatwave exposure. Increasing water velocity can reduce the diffusive boundary layer around kelp blades, leading to faster carbon and nitrogen uptake and increased productivity (Hurd et al., 1996; Wheeler, 1980). However, once nutrient uptake is saturated, further increases in water velocity do not drive increased growth and can cause a decrease in linear growth rates, as kelp may divert more resources toward blade thickening and strengthening to reduce the impacts of drag (Kregting et al., 2016; Peteiro et al., 2019). In the ambient‐light treatments, increasing water velocity might have allowed sporophytes to take up and store more carbon, nitrogen, and micronutrients, perhaps increasing their resilience to thermal stress. Conversely, kelp photosynthetic rates may have been constrained by low light availability in the shaded treatments, thus reducing their demand for carbon dioxide, while thermal stress could have reduced their tissue strength (Simonson et al., 2015). The variation in water velocity with depth in our experimental setup (see Figures [Link], [Link]) suggests that a strong shearing effect may have occurred in the tanks, putting further strain on the sporophyte blades. Hence, any positive effect of increased water velocity on nutrient uptake in these treatments was likely outweighed by the adverse impacts of rapid blade erosion due to increased drag forces.

Although we found some evidence that a combination of light limitation and fast water velocity could be harmful to kelp, other studies have shown contrasting trends. Water velocity did not modify the positive impact of light availability on growth rates of juvenile *Ecklonia radiata*, whereas recruitment was optimal under a combination of either saturating light and reduced water velocity, or reduced light and ambient water velocity (Tatsumi et al., 2021). New recruits and growing sporophytes have different light requirements (Graham et al., 1997), so interactions between light availability and other drivers would also be expected to vary between life‐history stages. In California, both reduced light availability and low water velocity independently reduced the growth rate of *Macrocystis pyrifera* meristematic tissue; no interactive effect was observed (Drobnitch et al., 2018). To date, few laboratory studies have investigated the combined effects of temperature and water movement on kelp physiology. Temperature can modify the effects of wave exposure on spore germination and the growth of newly emerged sporophytes in *M. pyrifera*, with spores from wave‐exposed sites in Chile having higher germination rates at elevated temperatures, and a combination of reduced wave exposure and low temperatures promoting faster growth (Buschmann et al., 2004). This result contrasts our findings, as we observed that temperature and water velocity had interactive effects on survival but not growth. Fast water motion can have both positive and negative impacts on kelp, with the nature of these impacts varying between life‐history stages (Beckley & Edwards, 2021; Graham et al., 1997; Hepburn et al., 2007), and this variation may modify the strength of any interactions between water velocity and other drivers.

Our results were consistent with previous studies in which warmer temperatures drove reduced blade growth in *Macrocystis pyrifera* sporophytes (Fernández et al., 2020; Mabin et al., 2019; Umanzor et al., 2021). Blade growth depends on several interrelated metabolic processes, all of which can be affected by temperature, which may make it difficult for kelp growth rates to acclimatize to increased temperatures (Fernández et al., 2020). Additionally, high temperatures can damage cellular structures and compromise tissue integrity in kelp blades (Simonson et al., 2015), allowing blades to erode rapidly (James et al., 2024), as they did in this experiment. Previous studies on Wellington's *M. pyrifera* population (Bunting et al., 2024), as well as populations from Chile (Rothäusler et al., 2009) and Tasmania (Fernández et al., 2020; Mabin et al., 2019), have observed severe decreases in sporophyte survival rates above 20°C. The upper thermal limit for survival in Tasmanian *M. pyrifera* sporophytes is between 24 and 27°C (Fernández et al., 2020). The 100% mortality in our 24°C treatments implies that the Wellington population has a similar upper limit. Among the heatwave simulations used in this experiment, the 20°C treatment is the most similar to maximum temperatures during previous MHWs that have occurred in the Wellington region and wider Aotearoa New Zealand, and a duration of 21 days is realistic (Behrens et al., 2022; Bunting et al., 2024, Figures [Link], [Link]). The 22 and 24°C treatments represent severe MHW scenarios that are currently rare within *M. pyrifera'*s distribution in New Zealand. However, these extreme MHWs could become more common under future warming conditions as average MHW intensities continue to increase (Behrens et al., 2022; Montie et al., 2023). Hence, the risk posed to *M. pyrifera* populations by MHWs is likely to increase in future decades.

The results of our experiment suggest that kelp populations in sites with ideal environmental conditions may be more resilient to marine heatwaves at sublethal temperatures when compared to stands in less optimal conditions. This experiment is not a perfect proxy for real‐world conditions: Our water velocity treatments did not replicate the full range and complexity of variation in hydrodynamics in subtidal habitats. We also simulated the impacts of sedimentation on light availability but not the direct physical impacts on kelp, which could be more severe under faster flow conditions (Devinny & Volse, 1978). Additionally, the kelp used in this experiment was sourced from a shallow‐water population, whereas the light treatments used in this experiment were chosen based on habitats ~ 10 m deep, where the effects of coastal darkening would typically be more pronounced (Desmond et al., 2015; Tait, 2019). Kelp populations from different water depths could differ in their tolerance to MHWs and light limitation (Almeida‐Saá et al., 2024). This experiment nonetheless demonstrates that water velocity and light availability can modify the impacts of MHWs on kelp sporophytes. It would therefore be worthwhile to consider other physical factors, including hydrodynamics and exposure to sediment pollution, when modeling how real‐world kelp populations could respond to marine heatwaves, as well as when designing management or restoration plans for these populations.

Kelp restoration projects have been developed in numerous countries, although research on kelp restoration has lagged behind other marine ecosystems globally (Eger et al., 2022). Several kelp restoration projects are currently ongoing in Aotearoa New Zealand, including *Macrocystis pyrifera* outplanting in Wellington and the South Island (Fisheries New Zealand, 2023). However, ocean warming poses a threat to the long‐term success of these restoration projects (Eger et al., 2022). Our findings suggest that one potential approach to mitigating this threat could involve outplanting kelp in sites with optimal combinations of environmental conditions, where kelp might be more resilient to MHWs and long‐term warming. Locations with good water clarity and fast tidal flows should be prioritized for *M. pyrifera* restoration. Wave‐sheltered sites could be more appropriate targets for restoration in regions where coastal darkening is a concern—for instance, near urban centers (Desmond et al., 2015) or in regions prone to heavy storms and flooding. It should be noted that light and hydrodynamics had a comparatively limited impact on sporophyte health when temperatures exceeded the 22°C threshold in our experiment. Hence, reducing greenhouse gas emissions should still be a priority to reduce the risk of sea temperatures surpassing this threshold and causing widespread damage to giant kelp populations.

## AUTHOR CONTRIBUTIONS

**Imogen Bunting:** Conceptualization (equal); data curation (lead); formal analysis (lead); investigation (lead); methodology (equal); visualization (lead); writing – original draft (lead); writing – review and editing (equal). **Laura Bornemann Santamaría:** Conceptualization (equal); investigation (lead); methodology (equal); writing – review and editing (equal). **Yun Yi Kok:** Resources (supporting); writing – review and editing (equal). **Erik C. Krieger:** Conceptualization (equal); methodology (equal); supervision (supporting); writing – review and editing (equal). **Julia C. Mullarney:** Investigation (supporting); methodology (supporting); resources (supporting); writing – review and editing (equal). **Roberta D'Archino:** Conceptualization (equal); methodology (equal); resources (equal); supervision (supporting); writing – review and editing (equal). **Christopher E. Cornwall:** Conceptualization (equal); funding acquisition (lead); methodology (equal); project administration (lead); resources (equal); supervision (lead); writing – review and editing (equal).

## FUNDING INFORMATION

This research was supported by funding from the Coastal People, Southern Skies Centre for Research Excellence project to CEC (E4280), a Rutherford Discovery Fellowship to CEC (VUW 1701), the Wellington Community Fund, the Eurofins Foundation, and the Clare Foundation.

## Supporting information


**Appendix S1.** Notes on water velocity measurements.

**Figure S1.** Location of the sori collection site, labeled in red, relative to the known distribution of *Macrocystis pyrifera* in New Zealand, shown in brown. *M. pyrifera* distribution data was obtained from GBIF.org (2025), using the R package rgbif (Chamberlain et al., 2024), and verified against Hay (1990).

**Figure S2.** Schematic of an experimental tank showing the positions at which water velocity was measured.

**Figure S3.** Flow speed (m · s^−1^) measured across a range of water depths (Height above bottom, HAB, in m) at the positions indicated in Figure S1, in both the one‐pump and two‐pump treatments.

**Figure S4.** *x*‐velocity (i.e., lengthwise velocity; m · s^−1^) measured across a range of water depths (Height above bottom, HAB, in m) at the positions indicated in Figure S1, in both the one‐pump and two‐pump treatments.

**Figure S5.** Mean flow speed (m · s^−1^) averaged across all six positions at each water depth, in both the one‐pump and two‐pump treatments.

**Table S1.** Mean relative growth rate (RGR) values, with standard error, of *Macrocystis pyrifera* sporophytes under each individual temperature, light, and water velocity treatment, each combination of light and water velocity treatments, and each combination of temperature, light, and water velocity treatments, for each experimental phase.

**Table S2.** Mean effective quantum yield (*F*_v_′/*F*_m_′) values, with standard error, of *Macrocystis pyrifera* sporophytes under each individual temperature, light, and water velocity treatment, each combination of light and water velocity treatments, and each combination of temperature, light, and water velocity treatments, for each experimental phase.

**Table S3.** Wet weight and calculated RGR values of *Macrocystis pyrifera* sporophytes during each experimental phase.

**Table S4.** Effective quantum yield (*F*_v_′/*F*_m_′) values of *Macrocystis pyrifera* sporophytes during each experimental phase.

**Table S5.** Temperature (T), pH_T_, and dissolved oxygen (DO) measurements taken in the experimental tanks, water baths, and header tanks throughout the experimental period.

**Table S6.** Ammonium (NH_4_^+^), total NO_x_, nitrite (NO_2_^−^), and nitrate (NO_3_^−^) concentrations (μg · L^−1^) of water samples taken from the experimental tanks and headers throughout the experimental period.

## Data Availability

Raw data for sporophyte growth and effective quantum yield are provided in the Tables S3 and S4, along with temperature, pH, dissolved oxygen, and nitrogen content data from the experimental tanks (Tables S5 and S6).
