# Supplementary material for: Light limitation and water velocity modify the impacts of simulated marine heatwaves on juvenile giant kelp
Source: J Phycol. 2025 Jul 18;61(5):1173–94. doi: 10.1111/jpy.70054 (PMC12547647; doi:10.1111/jpy.70054)
Supplement: Supplementary file 1 — Appendix S1. Notes on water velocity measurements. [file JPY-61-1173-s009.docx]

**Methods**

To provide an estimation of typical flow speeds and the spatial variability of velocities in the experiments with one and two pumps, measurements were captured using a profiling Acoustic Doppler Velocimeter (ADV – a 10 MHz Nortek Vectrino Profiler (Nortek, Rud, Norway)) in an experiment without kelp. Water depths were ~9.5 cm, and the pump outlets were at ~7.5 cm above the bed. The instrument measures a short (up to 3 cm long) profile of three components of velocity with 1 mm vertical resolution at 50 Hz. For both one and two pump treatments, six horizontal positions throughout the tank were measured (*x* = 8.75, 11.75, and 14.75 cm, and *y* = 5.5 and 10 cm, where *x* = *y* = 0 corresponds to the bottom left corner; Figure S1). Additionally, at each horizontal location, the ADV was moved through six vertical locations with profiles starting at the bed to capture velocities at varying heights. Data was recorded for one minute at each position. Owing to the blanking distance between the transducer and the first measurement, data were only able to be resolved in the bottom ~4 cm of the tank. A dye release close to the surface was also used to provide very approximate estimates of the near surface flows.

To minimize noise from acoustic reflections in the small tank, very stringent quality control measures were implemented. Low quality data (correlations < 95%, and SNR < 15 or 25 dB in the one and two pump cases, respectively) were removed. Additionally, all data from a vertical bin with less than 25% of measurements classified as ‘good’ were discarded. These criteria only retained data in the ‘sweet spot’ of the profile (see Brand et al., 2016), and the remaining velocities from overlapping heights were generally in reasonable agreement, and linearly interpolated to provide a continuous equally spaced profile in the lower half of the tank.

**Results**

Raw water velocity data are available from the corresponding author on request. Flow speeds are shown in Figure S2. In both experiments, flow speeds away from the bed were similar – around 5–6 cm s^-1^. However, the speeds in the two-pump case were generally slightly faster. Of note, there was a region of faster flow speeds near the bed at position 3 in the single-pump case. This region corresponds to a lower layer return flow being generated by the surface flow hitting the end wall directly opposite the pump (in the along-tank direction) and being forced downwards and back towards the pump (Figure S3). In the two-pump case, such return flow is generated at both ends of the tank (i.e. positions 3 and 4), thus the spatial average of the near bed flow is also larger in this case. Hence, flows were significantly more vertically sheared during the two-pump experiment, and closer to depth-uniform in the one pump experiment. The dye releases also supported these conclusions – in the one-pump case, flows in the surface layer were 0.1–0.15 m s^-1^ on the side of the tank with the pump, and ~0.03–0.05 m s^-1^ on the other side; whereas in the two-pump case, surface flows were ~0.1–0.15 m s^-1^ on both sides (directed away from the pump).

**References**

Brand, A., Noss, C., Dinkel, C. and Holzner, M. (2016). High-resolution measurements of turbulent flow close to the sediment–water interface using a bistatic acoustic profiler. *Journal of Atmospheric and Oceanic Technology* 33: 769–788. doi: 10.1175/JTECH-D-15-0152.1
