# Supplementary figures and images for: Light limitation and water velocity modify the impacts of simulated marine heatwaves on juvenile giant kelp
Source: J Phycol. 2025 Jul 18;61(5):1173–94. doi: 10.1111/jpy.70054 (PMC12547647; doi:10.1111/jpy.70054)

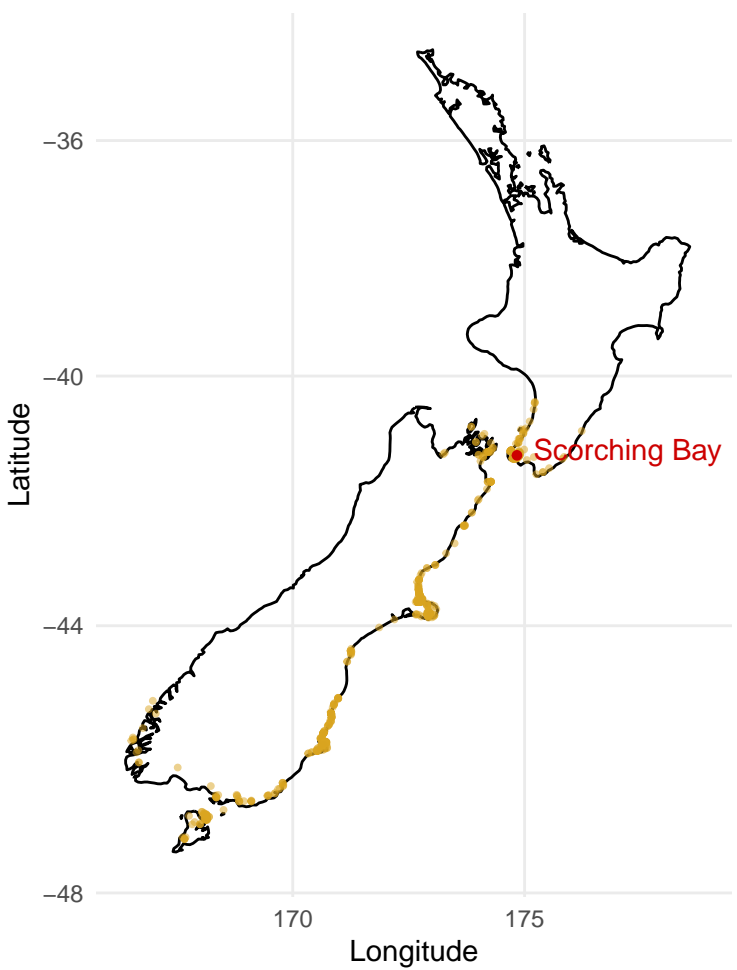

Supplement: Supplementary file 2 — Figure S1. Location of the sori collection site, labeled in red, relative to the known distribution of Macrocystis pyrifera in New Zealand, shown in brown. M. pyrifera distribution data was obtained from GBIF.org (2025), using the R package rgbif (Chamberlain et al., 2024), and verified against Hay (1990). [file JPY-61-1173-s008.pdf]

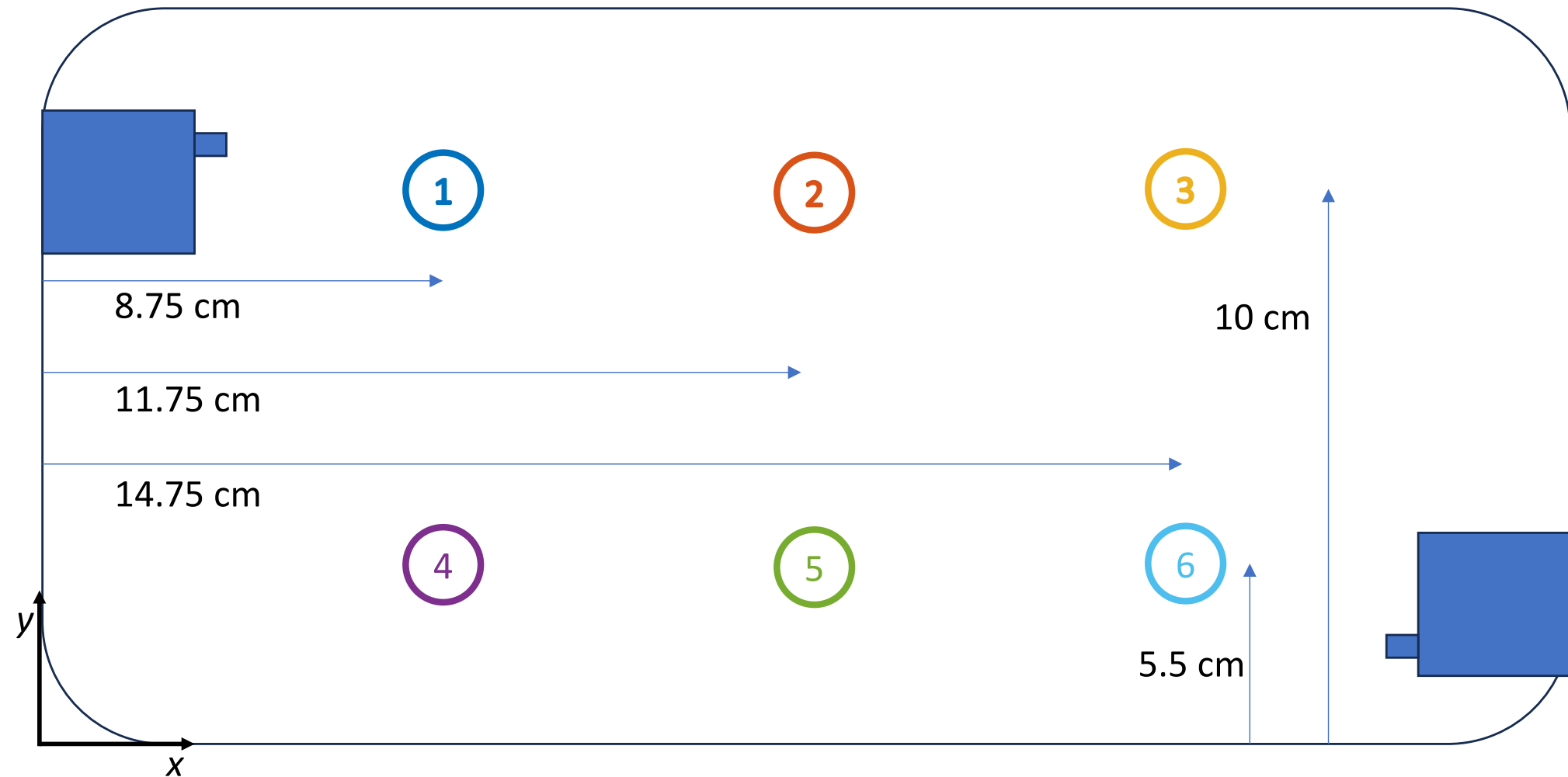

Supplement: Supplementary file 3 — Figure S2. Schematic of an experimental tank showing the positions at which water velocity was measured. [file JPY-61-1173-s007.pdf]

**One pump**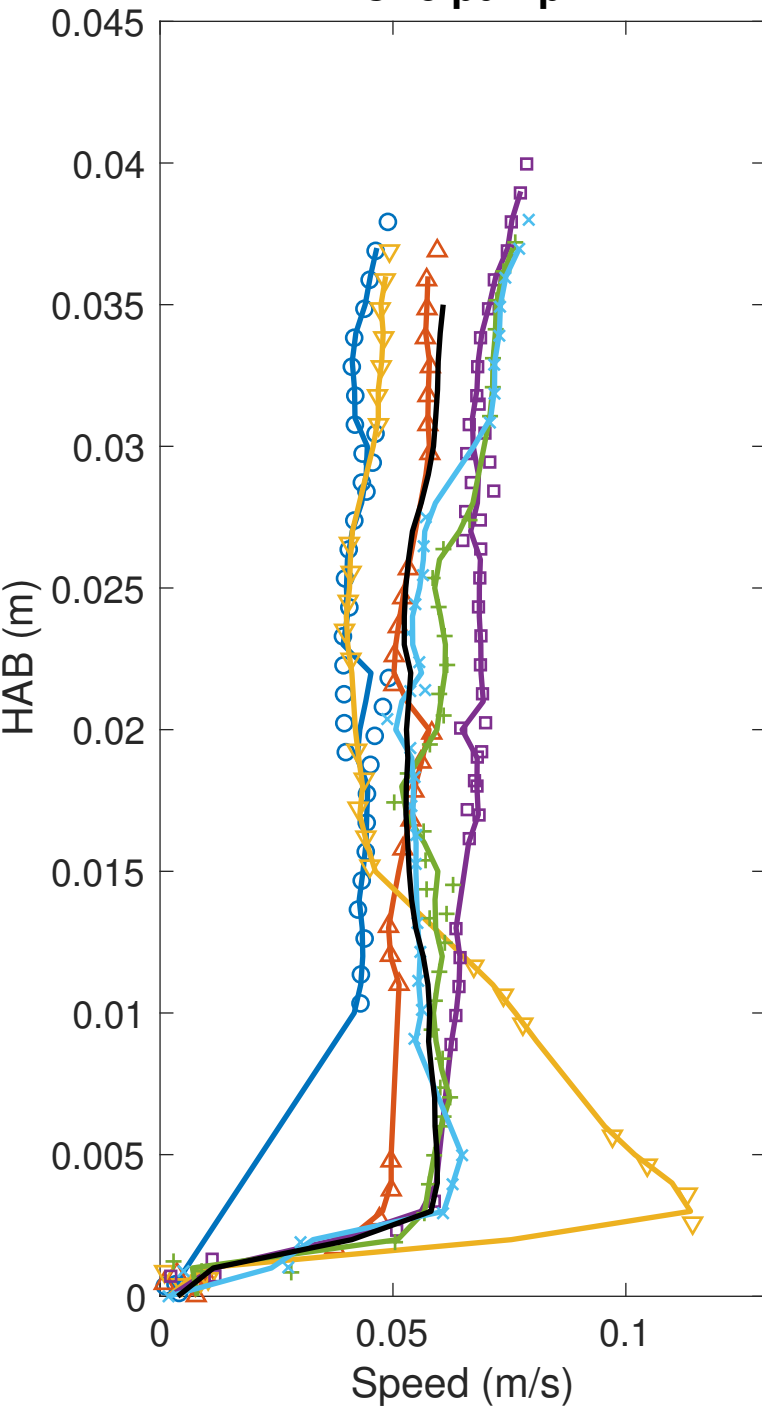**Two pumps**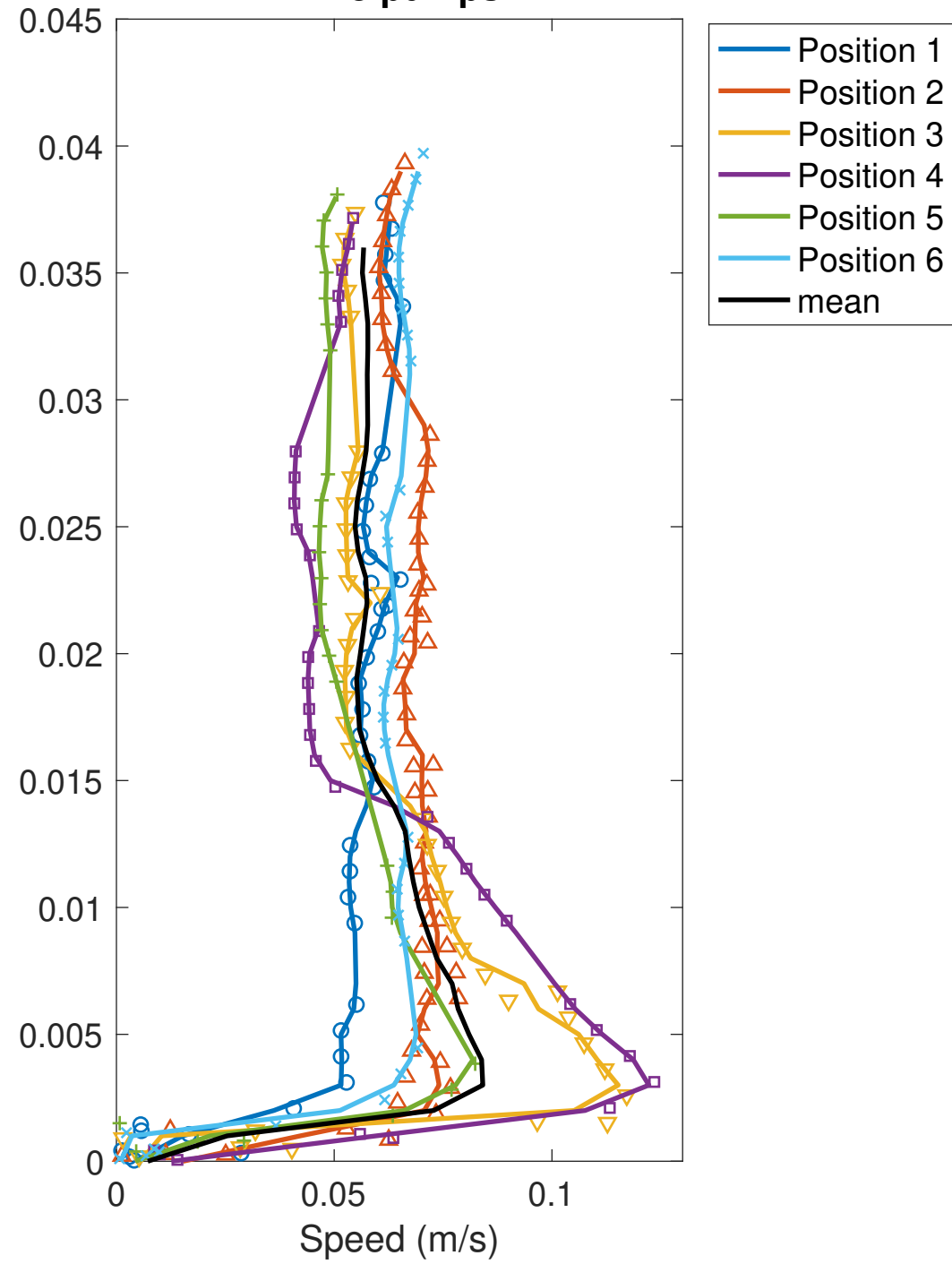

Supplement: Supplementary file 4 — Figure S3. Flow speed (m · s−1) measured across a range of water depths (Height above bottom, HAB, in m) at the positions indicated in Figure S1, in both the one‐pump and two‐pump treatments. [file JPY-61-1173-s003.pdf]

**One pump**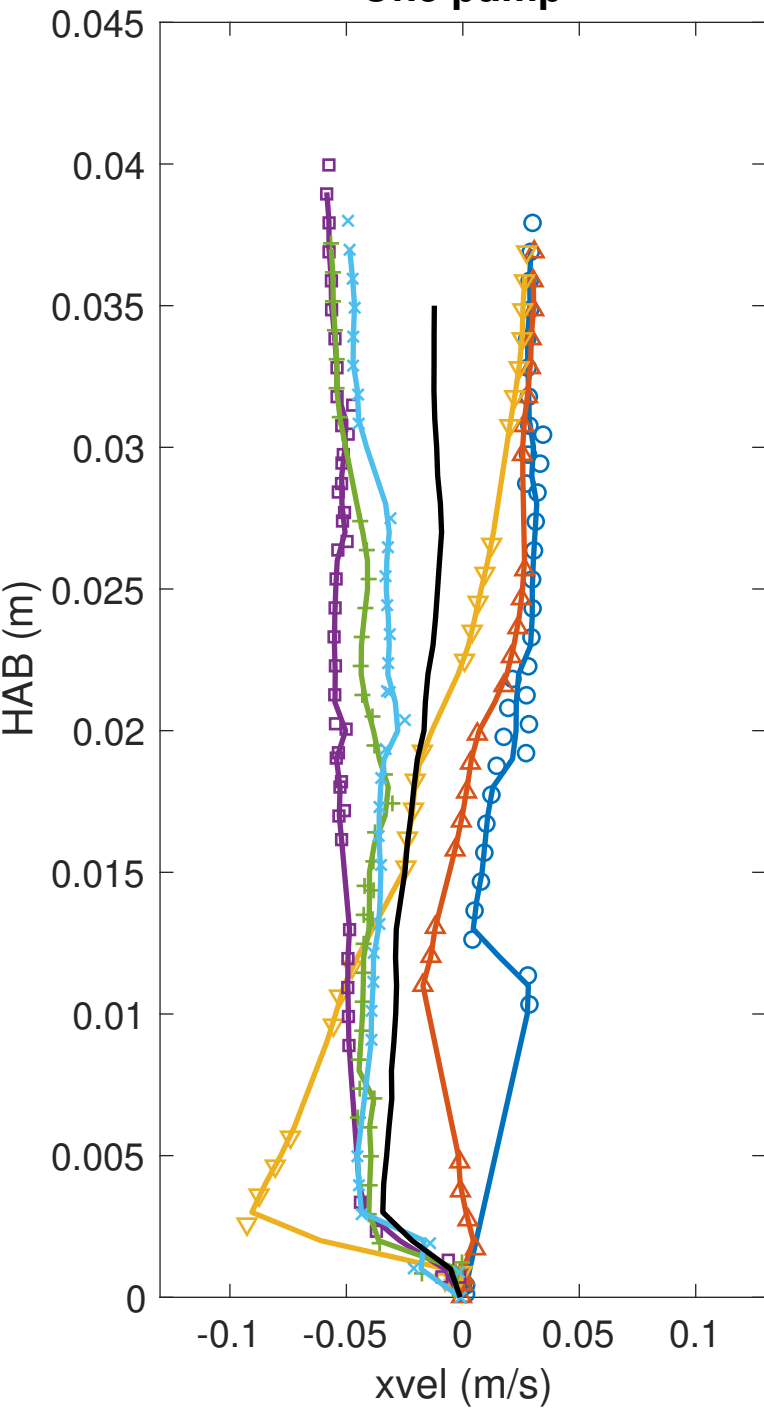**Two pumps**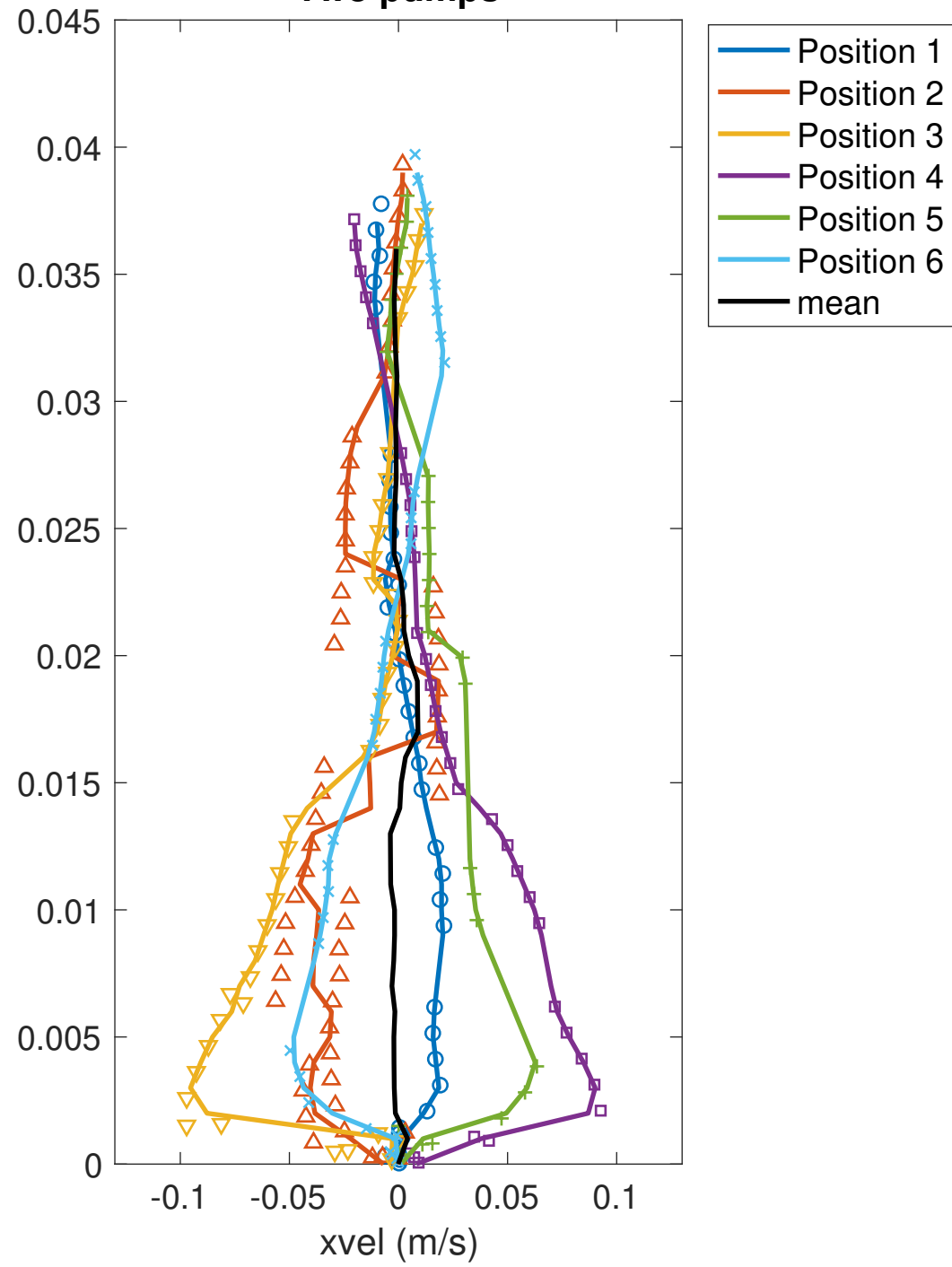

Supplement: Supplementary file 5 — Figure S4. x‐velocity (i.e., lengthwise velocity; m · s−1) measured across a range of water depths (Height above bottom, HAB, in m) at the positions indicated in Figure S1, in both the one‐pump and two‐pump treatments. [file JPY-61-1173-s001.pdf]

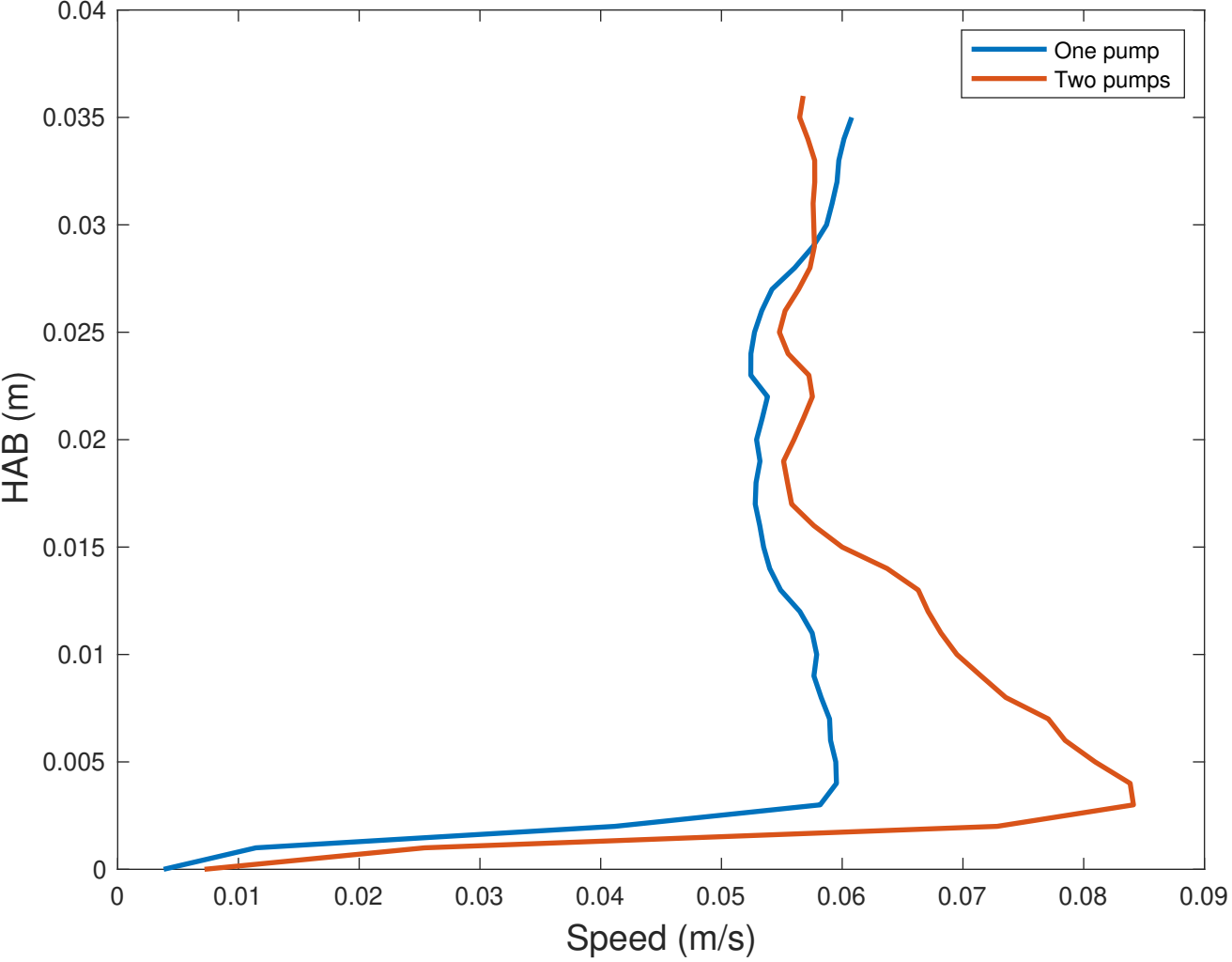

Supplement: Supplementary file 6 — Figure S5. Mean flow speed (m · s−1) averaged across all six positions at each water depth, in both the one‐pump and two‐pump treatments. [file JPY-61-1173-s010.pdf]
