# Supplementary material for: Light limitation and water velocity modify the impacts of simulated marine heatwaves on juvenile giant kelp
Source: J Phycol. 2025 Jul 18;61(5):1173–94. doi: 10.1111/jpy.70054 (PMC12547647; doi:10.1111/jpy.70054)
Supplement: Supplementary file 8 — Table S2. Mean effective quantum yield (F v′/F m′) values, with standard error, of Macrocystis pyrifera sporophytes under each individual temperature, light, and water velocity treatment, each combination of light and water velocity treatments, and each combination of temperature, light, and water velocity treatments, for each experimental phase. [file JPY-61-1173-s012.docx]

|  | Timepoint | Treatment | Mean *F*_v’_/*F*_m’_ ± standard error |
| --- | --- | --- | --- |
| Temperature (°C) | Acclimation | 16 | 0.722 ± 0.006 |
|  |  | 20 | 0.714 ± 0.011 |
|  |  | 22 | 0.718 ± 0.009 |
|  |  | 24 | 0.713 ± 0.005 |
|  | Heatwave | 16 | 0.722 ± 0.006 |
|  |  | 20 | 0.709 ± 0.016 |
|  |  | 22 | 0.665 ± 0.037 |
|  |  | 24 | 0.608 |
|  | Recovery | 16 | 0.708 ± 0.007 |
|  |  | 20 | 0.697 ± 0.014 |
|  |  | 22 | 0.684 ± 0.029 |
| Light | Acclimation | Light | 0.715 ± 0.006 |
|  |  | Shade | 0.719 ± 0.005 |
|  | Heatwave | Light | 0.711 ± 0.011 |
|  |  | Shade | 0.699 ± 0.014 |
|  | Recovery | Light | 0.704 ± 0.009 |
|  |  | Shade | 0.695 ± 0.012 |
| Water velocity | Acclimation | Fast | 0.715 ± 0.006 |
|  |  | Slow | 0.718 ± 0.006 |
|  | Heatwave | Fast | 0.707 ± 0.013 |
|  |  | Slow | 0.706 ± 0.013 |
|  | Recovery | Fast | 0.695 ± 0.011 |
|  |  | Slow | 0.706 ± 0.009 |
| Light × water velocity | Acclimation | Light × fast | 0.711 ± 0.008 |
|  |  | Light × slow | 0.718 ± 0.010 |
|  |  | Shade × fast | 0.720 ± 0.008 |
|  |  | Shade × slow | 0.718 ± 0.006 |
|  | Heatwave | Light × fast | 0.704 ± 0.017 |
|  |  | Light × slow | 0.719 ± 0.016 |
|  |  | Shade × fast | 0.715 ± 0.014 |
|  |  | Shade × slow | 0.692 ± 0.020 |
|  | Recovery | Light × fast | 0.695 ± 0.012 |
|  |  | Light × slow | 0.722 ± 0.008 |
|  |  | Shade × fast | 0.696 ± 0.029 |
|  |  | Shade × slow | 0.694 ± 0.013 |
| Temperature × light × water velocity | Acclimation | 16 × light × fast | 0.697 ± 0.008 |
|  |  | 16 × light × slow | 0.731 ± 0.013 |
|  |  | 16 × shade × fast | 0.740 ± 0.004 |
|  |  | 16 × shade × slow | 0.726 ± 0.009 |
|  |  | 20 × light × fast | 0.726 ± 0.011 |
|  |  | 20 × light × slow | 0.698 ± 0.039 |
|  |  | 20 × shade × fast | 0.725 ± 0.011 |
|  |  | 20 × shade × slow | 0.713 ± 0.017 |
|  |  | 22 × light × fast | 0.719 ± 0.025 |
|  |  | 22 × light × slow | 0.729 ± 0.011 |
|  |  | 22 × shade × fast | 0.709 ± 0.021 |
|  |  | 22 × shade × slow | 0.716 ± 0.022 |
|  |  | 24 × light × fast | 0.702 ± 0.015 |
|  |  | 24 × light × slow | 0.716 ± 0.010 |
|  |  | 24 × shade × fast | 0.715 ± 0.012 |
|  |  | 24 × shade × slow | 0.719 ± 0.007 |
|  | Heatwave | 16 × light × fast | 0.730 ± 0.009 |
|  |  | 16 × light × slow | 0.736 ± 0.004 |
|  |  | 16 × shade × fast | 0.716 ± 0.020 |
|  |  | 16 × shade × slow | 0.706 ± 0.015 |
|  |  | 20 × light × fast | 0.721 ± 0.019 |
|  |  | 20 × light × slow | 0.745 ± 0.002 |
|  |  | 20 × shade × fast | 0.710 |
|  |  | 20 × shade × slow | 0.678 ± 0.039 |
|  |  | 22 × light × fast | 0.648 ± 0.047 |
|  |  | 22 × light × slow | 0.715 |
|  |  | 24 × light × slow | 0.608 |
|  | Recovery | 16 × light × fast | 0.696 ± 0.013 |
|  |  | 16 × light × slow | 0.717 ± 0.011 |
|  |  | 16 × shade × fast | 0.730 ± 0.003 |
|  |  | 16 × shade × slow | 0.699 ± 0.014 |
|  |  | 20 × light × fast | 0.684 ± 0.029 |
|  |  | 20 × light × slow | 0.733 ± 0.008 |
|  |  | 20 × shade × fast | 0.712 |
|  |  | 20 × shade × slow | 0.690 ± 0.025 |
|  |  | 22 × light × fast | 0.708 ± 0.021 |
|  |  | 22 × shade × fast | 0.610 |
