# Supplementary material for: Light limitation and water velocity modify the impacts of simulated marine heatwaves on juvenile giant kelp
Source: J Phycol. 2025 Jul 18;61(5):1173–94. doi: 10.1111/jpy.70054 (PMC12547647; doi:10.1111/jpy.70054)
Supplement: Supplementary file 9 — Table S3. Wet weight and calculated RGR values of Macrocystis pyrifera sporophytes during each experimental phase. [file JPY-61-1173-s011.docx]

| Tank | Light level | Water velocity | Temp (°C) | Specimen | 09/02/2023 | | 02/03/2023 | | | 25/03/2023 | | | 13/04/2023 | |
| --- | --- | --- | --- | --- | --- | --- | --- | --- | --- | --- | --- | --- | --- | --- |
|  |  |  |  |  | **Weight (g)** | **Weight (g)** | | **RGR (% day^-1^)** | **Weight (g)** | | **RGR (% day^-1^)** | **Weight (g)** | | **RGR (% day^-1^)** |
| A1 | Light | Fast | 22 | A11 | 0.19 | 0.46 | | 4.21 | 0.29 | | -2.01 | 0.53 | | 3.17 |
|  |  |  |  | A12 | 0.01 | 0.08 | | 9.90 | 0.03 | | -4.26 | 0.16 | | 8.81 |
| A2 | Light | Slow | 22 | A21 | 0.24 | 0.21 | | -0.64 | X | | X | X | | X |
|  |  |  |  | A22 | 0.09 | 0.21 | | 4.03 | X | | X | X | | X |
| A3 | Shade | Slow | 22 | A31 | 0.26 | X | | X | X | | X | X | | X |
|  |  |  |  | A32 | 0.23 | 0.21 | | -0.43 | X | | X | X | | X |
| A4 | Shade | Fast | 22 | A41 | 0.2 | 0.22 | | 0.45 | X | | X | X | | X |
|  |  |  |  | A42 | 0.21 | 0.18 | | -0.73 | X | | X | X | | X |
| B1 | Shade | Slow | 16 | B11 | 0.16 | 0.12 | | -1.37 | 0.08 | | -1.76 | 0.15 | | 3.31 |
|  |  |  |  | B12 | 0.17 | 0.14 | | -0.92 | 0.1 | | -1.46 | 0.19 | | 3.38 |
| B2 | Shade | Fast | 16 | B21 | 0.43 | 0.37 | | -0.72 | 0.39 | | 0.23 | 0.68 | | 2.93 |
|  |  |  |  | B22 | 0.25 | 0.22 | | -0.61 | 0.37 | | 2.26 | 0.47 | | 1.26 |
| B3 | Light | Fast | 16 | B31 | 0.32 | 0.38 | | 0.82 | 0.37 | | -0.12 | 0.38 | | 0.14 |
|  |  |  |  | B32 | 0.1 | 0.16 | | 2.24 | 0.17 | | 0.26 | 0.32 | | 3.33 |
| B4 | Light | Slow | 16 | B41 | 0.16 | 0.28 | | 2.66 | 0.25 | | -0.49 | 0.75 | | 5.78 |
|  |  |  |  | B42 | 0.21 | 0.36 | | 2.57 | 0.3 | | -0.79 | 0.42 | | 1.77 |
| C1 | Light | Fast | 20 | C11 | 0.06 | 0.01 | | -8.53 | 0.04 | | 6.03 | 0.17 | | 7.62 |
|  |  |  |  | C12 | 0.04 | 0.07 | | 2.66 | 0.06 | | -0.67 | X | | X |
| C2 | Light | Slow | 20 | C21 | 0.18 | 0.35 | | 3.17 | 0.39 | | 0.47 | 0.51 | | 1.41 |
|  |  |  |  | C22 | 0.07 | 0.18 | | 4.50 | 0.09 | | -3.01 | 0.24 | | 5.16 |
| C3 | Shade | Slow | 20 | C31 | 0.07 | 0.08 | | 0.64 | 0.05 | | -2.04 | 0.07 | | 1.77 |
|  |  |  |  | C32 | 0.23 | 0.38 | | 2.39 | 0.08 | | -6.77 | 0.15 | | 3.31 |
| C4 | Shade | Fast | 20 | C41 | 0.35 | X | | X | X | | X | X | | X |
|  |  |  |  | C42 | 0.14 | 0.11 | | -1.15 | 0.1 | | -0.41 | 0.17 | | 2.79 |
| D1 | Shade | Slow | 24 | D11 | 0.09 | 0.11 | | 0.96 | X | | X | X | | X |
|  |  |  |  | D12 | 0.34 | 0.43 | | 1.12 | X | | X | X | | X |
| D2 | Shade | Fast | 24 | D21 | 0.13 | 0.11 | | -0.80 | X | | X | X | | X |
|  |  |  |  | D22 | 0.64 | 0.68 | | 0.29 | 0.27 | | -4.02 | X | | X |
| D3 | Light | Fast | 24 | D31 | 0.2 | 0.41 | | 3.42 | X | | X | X | | X |
|  |  |  |  | D32 | 0.1 | 0.14 | | 1.60 | 0.05 | | -4.48 | 0.11 | | 4.15 |
| D4 | Light | Slow | 24 | D41 | 0.27 | 0.5 | | 2.93 | 0.09 | | -7.46 | 0.17 | | 3.35 |
|  |  |  |  | D42 | 0.12 | 0.15 | | 1.06 | X | | X | X | | X |
| E1 | Shade | Fast | 20 | E11 | 0.17 | 0.26 | | 2.02 | X | | X | X | | X |
|  |  |  |  | E12 | 0.15 | X | | X | X | | X | X | | X |
| E2 | Shade | Slow | 20 | E21 | 0.32 | 0.19 | | -2.48 | 0.18 | | -0.24 | 0.2 | | 0.55 |
|  |  |  |  | E22 | 0.08 | 0.14 | | 2.66 | 0.14 | | 0.00 | 0.14 | | 0.00 |
| E3 | Light | Slow | 20 | E31 | 0.04 | 0.08 | | 3.30 | X | | X | X | | X |
|  |  |  |  | E32 | 0.16 | 0.14 | | -0.64 | X | | X | X | | X |
| E4 | Light | Fast | 20 | E41 | 0.22 | 0.31 | | 1.63 | 0.35 | | 0.53 | 0.46 | | 1.44 |
|  |  |  |  | E42 | 0.09 | 0.18 | | 3.30 | 0.2 | | 0.46 | 0.21 | | 0.26 |
| F1 | Light | Fast | 24 | F11 | 0.19 | 0.14 | | -1.45 | 0.17 | | 0.84 | 0.11 | | -2.29 |
|  |  |  |  | F12 | 0.24 | 0.22 | | -0.41 | 0.08 | | -4.40 | 0.12 | | 2.13 |
| F2 | Light | Slow | 24 | F21 | 0.31 | 0.59 | | 3.06 | 0.17 | | -5.41 | 0.16 | | -0.32 |
|  |  |  |  | F22 | 0.08 | 0.08 | | 0.00 | X | | X | X | | X |
| F3 | Shade | Slow | 24 | F31 | 0.12 | 0.15 | | 1.06 | 0.05 | | -4.78 | 0.06 | | 0.96 |
|  |  |  |  | F32 | 0.19 | 0.13 | | -1.81 | 0.1 | | -1.14 | 0.08 | | -1.17 |
| F4 | Shade | Fast | 24 | F41 | 0.49 | 0.38 | | -1.21 | 0.13 | | -4.66 | 0.07 | | -3.26 |
|  |  |  |  | F42 | 0.14 | 0.23 | | 2.36 | X | | X | X | | X |
| G1 | Shade | Slow | 22 | G11 | 0.1 | 0.14 | | 1.60 | 0.09 | | -1.92 | 0.09 | | 0.00 |
|  |  |  |  | G12 | 0.13 | 0.13 | | 0.00 | 0.09 | | -1.60 | X | | X |
| G2 | Shade | Fast | 22 | G21 | 0.08 | 0.07 | | -0.64 | 0.08 | | 0.58 | 0.09 | | 0.62 |
|  |  |  |  | G22 | 0.13 | 0.24 | | 2.92 | 0.08 | | -4.78 | 0.15 | | 3.31 |
| G3 | Light | Fast | 22 | G31 | 0.33 | 0.47 | | 1.68 | 0.21 | | -3.50 | 0.17 | | -1.11 |
|  |  |  |  | G32 | 0.04 | 0.09 | | 3.86 | X | | X | X | | X |
| G4 | Light | Slow | 22 | G41 | 0.2 | 0.37 | | 2.93 | 0.42 | | 0.55 | 0.2 | | -3.90 |
|  |  |  |  | G42 | 0.12 | 0.15 | | 1.06 | 0.06 | | -3.98 | 0.14 | | 4.46 |
| H1 | Light | Fast | 16 | H11 | 0.18 | 0.29 | | 2.27 | 0.42 | | 1.61 | 0.64 | | 2.22 |
|  |  |  |  | H12 | 0.17 | 0.17 | | 0.00 | 0.18 | | 0.25 | 0.25 | | 1.73 |
| H2 | Light | Slow | 16 | H21 | 0.06 | 0.07 | | 0.73 | 0.09 | | 1.09 | 0.22 | | 4.70 |
|  |  |  |  | H22 | 0.09 | 0.14 | | 2.10 | 0.15 | | 0.30 | 0.31 | | 3.82 |
| H3 | Shade | Slow | 16 | H31 | 0.2 | 0.31 | | 2.09 | 0.29 | | -0.29 | 0.2 | | -1.96 |
|  |  |  |  | H32 | 0.15 | 0.17 | | 0.60 | 0.15 | | -0.54 | 0.17 | | 0.66 |
| H4 | Shade | Fast | 16 | H41 | 0.13 | X | | X | X | | X | X | | X |
|  |  |  |  | H42 | 0.31 | 0.22 | | -1.63 | 0.22 | | 0.00 | 0.19 | | -0.77 |
