# Supplementary material for: Light limitation and water velocity modify the impacts of simulated marine heatwaves on juvenile giant kelp
Source: J Phycol. 2025 Jul 18;61(5):1173–94. doi: 10.1111/jpy.70054 (PMC12547647; doi:10.1111/jpy.70054)
Supplement: Supplementary file 10 — Table S4. Effective quantum yield (F v′/F m′) values of Macrocystis pyrifera sporophytes during each experimental phase. [file JPY-61-1173-s002.docx]

| Tank | Light level | Water velocity | Temp (°C) | Specimen | *F*_v’_/*F*_m’_ | | |
| --- | --- | --- | --- | --- | --- | --- | --- |
|  |  |  |  |  | **01/03/2023**  **Acclimation** | **22/03/2023**  **Heatwave** | **12/04/2023**  **Recovery** |
| A1 | Light | Fast | 22 | A11 | 0.744 | 0.738 | 0.745 |
|  |  |  |  | A12 | 0.758 | 0.625 | 0.707 |
| A2 | Light | Slow | 22 | A21 | 0.744 | X | X |
|  |  |  |  | A22 | 0.747 | X | X |
| A3 | Shade | Slow | 22 | A31 | X | X | X |
|  |  |  |  | A32 | 0.759 | X | X |
| A4 | Shade | Fast | 22 | A41 | 0.745 | X | X |
|  |  |  |  | A42 | 0.650 | X | X |
| B1 | Shade | Slow | 16 | B11 | 0.701 | 0.668 | 0.672 |
|  |  |  |  | B12 | 0.732 | 0.722 | 0.716 |
| B2 | Shade | Fast | 16 | B21 | 0.733 | 0.735 | 0.733 |
|  |  |  |  | B22 | 0.743 | 0.737 | 0.727 |
| B3 | Light | Fast | 16 | B31 | 0.718 | 0.746 | 0.686 |
|  |  |  |  | B32 | 0.677 | 0.709 | 0.716 |
| B4 | Light | Slow | 16 | B41 | 0.744 | 0.736 | 0.722 |
|  |  |  |  | B42 | 0.748 | 0.739 | 0.737 |
| C1 | Light | Fast | 20 | C11 | 0.750 | 0.674 | 0.605 |
|  |  |  |  | C12 | 0.714 | 0.706 | 0.681 |
| C2 | Light | Slow | 20 | C21 | 0.746 | 0.747 | 0.740 |
|  |  |  |  | C22 | 0.755 | 0.743 | 0.725 |
| C3 | Shade | Slow | 20 | C31 | 0.728 | 0.606 | 0.663 |
|  |  |  |  | C32 | 0.728 | 0.616 | 0.634 |
| C4 | Shade | 2 | 20 | C41 | X | X | X |
|  |  | Fast |  | C42 | 0.714 | 0.71 | 0.712 |
| D1 | Shade | Slow | 24 | D11 | 0.705 | X | X |
|  |  |  |  | D12 | 0.736 | X | X |
| D2 | Shade | Fast | 24 | D21 | 0.721 | X | X |
|  |  |  |  | D22 | 0.720 | X | X |
| D3 | Light | Fast | 24 | D31 | 0.729 | X | X |
|  |  |  |  | D32 | 0.721 | X | X |
| D4 | Light | Slow | 24 | D41 | 0.730 | X | X |
|  |  |  |  | D42 | 0.706 | X | X |
| E1 | Shade | Fast | 20 | E11 | 0.736 | X | X |
|  |  |  |  | E12 | X | X | X |
| E2 | Shade | Slow | 20 | E21 | 0.663 | 0.750 | 0.726 |
|  |  |  |  | E22 | 0.732 | 0.738 | 0.736 |
| E3 | Light | Slow | 20 | E31 | 0.705 | X | X |
|  |  |  |  | E32 | 0.586 | X | X |
| E4 | Light | Fast | 20 | E41 | 0.738 | 0.753 | 0.722 |
|  |  |  |  | E42 | 0.701 | 0.752 | 0.726 |
| F1 | Light | Fast | 24 | F11 | 0.662 | X | X |
|  |  |  |  | F12 | 0.696 | X | X |
| F2 | Light | Slow | 24 | F21 | 0.737 | 0.608 | X |
|  |  |  |  | F22 | 0.692 | X | X |
| F3 | Shade | Slow | 24 | F31 | 0.714 | X | X |
|  |  |  |  | F32 | 0.719 | X | X |
| F4 | Shade | Fast | 24 | F41 | 0.738 | X | X |
|  |  |  |  | F42 | 0.680 | X | X |
| G1 | Shade | Slow | 22 | G11 | 0.694 | X | X |
|  |  |  |  | G12 | 0.695 | X | X |
| G2 | Shade | Fast | 22 | G21 | 0.713 | X | X |
|  |  |  |  | G22 | 0.727 | X | 0.61 |
| G3 | Light | Fast | 22 | G31 | 0.727 | 0.581 | 0.673 |
|  |  |  |  | G32 | 0.645 | X | X |
| G4 | Light | Slow | 22 | G41 | 0.727 | 0.715 | X |
|  |  |  |  | G42 | 0.697 | X | X |
| H1 | Light | Fast | 16 | H11 | 0.699 | 0.743 | 0.717 |
|  |  |  |  | H12 | 0.694 | 0.721 | 0.665 |
| H2 | Light | Slow | 16 | H21 | 0.692 | 0.726 | 0.722 |
|  |  |  |  | H22 | 0.739 | 0.743 | 0.686 |
| H3 | Shade | Slow | 16 | H31 | 0.741 | 0.736 | 0.729 |
|  |  |  |  | H32 | 0.728 | 0.697 | 0.678 |
| H4 | Shade | Fast | 16 | H41 | X | X | X |
|  |  |  |  | H42 | 0.745 | 0.676 |  |
