# Supplementary material for: Light limitation and water velocity modify the impacts of simulated marine heatwaves on juvenile giant kelp
Source: J Phycol. 2025 Jul 18;61(5):1173–94. doi: 10.1111/jpy.70054 (PMC12547647; doi:10.1111/jpy.70054)
Supplement: Supplementary file 11 — Table S5. Temperature (T), pHT, and dissolved oxygen (DO) measurements taken in the experimental tanks, water baths, and header tanks throughout the experimental period. [file JPY-61-1173-s006.docx]

| Date | 15/02/23 | | | 22/02/23 | | | 01/03/23 | | | 17/03/23 | | | 24/03/23 | | | 05/04/2023 | | | 12/04/23 | | |
| --- | --- | --- | --- | --- | --- | --- | --- | --- | --- | --- | --- | --- | --- | --- | --- | --- | --- | --- | --- | --- | --- |
|  | T (°C) | pH | DO (%) | T (°C) | pH | DO (%) | T (°C) | pH | DO (%) | T (°C) | pH | DO (%) | T (°C) | pH | DO (%) | T (°C) | pH | DO (%) | T (°C) | pH | DO (%) |
| A1 | 17.1 | 7.94 | 104.3 | 17.5 | 7.98 | 102 | 16.8 | 7.97 | 101.5 | 21.6 | 7.93 | 106.8 | 16.1 | 7.94 | 101.2 | 16.6 | 8.05 | 107.5 | 16.7 | 8.04 | 104.1 |
| A2 | 16.9 | 7.95 | 103.3 | 17.9 | 7.97 | 103.1 | 16.6 | 7.97 | 104 | 21.1 | 7.94 | 112.5 | 15.8 | 7.95 | 103.5 | 16.2 | 8.04 | 106 | 16.2 | 8.02 | 101.6 |
| A3 | 17.1 | 7.94 | 104 | 17.2 | 7.99 | 100 | 16.3 | 7.98 | 101.7 | 21.5 | 7.90 | 104.2 | 15.8 | 7.94 | 100.4 | 15.9 | 8.04 | 105.6 | 16.2 | 8.03 | 103 |
| A4 | 16.8 | 7.95 | 102.1 | 17.9 | 7.98 | 101.6 | 16.6 | 7.98 | 103.2 | 21.4 | 7.89 | 102.6 | 15.9 | 7.94 | 101.5 | 16.2 | 8.03 | 104.6 | 16.2 | 8.02 | 101.6 |
| Wb A | 17.8 | 7.93 | 103.5 | 17.9 | 7.98 | 101.1 | 16.6 | 7.98 | 101.5 | 27.2 | 7.84 | 111 | 16.1 | 7.95 | 101.8 | 16.5 | 8.06 | 109.7 | 16.4 | 8.03 | 103.3 |
| B1 | 16.7 | 7.97 | 105.4 | 17.6 | 8.00 | 101.3 | 16.7 | 7.97 | 101.6 | 17.5 | 7.97 | 102.3 | 15.9 | 7.96 | 102.5 | 16.3 | 8.05 | 110.4 | 16.2 | 8.03 | 104.4 |
| B2 | 15.9 | 7.98 | 101.6 | 17.9 | 7.99 | 102.6 | 16.4 | 7.98 | 103.4 | 17.1 | 7.99 | 102.9 | 15.9 | 7.96 | 102.6 | 16.5 | 8.06 | 108.4 | 16.1 | 8.02 | 101.5 |
| B3 | 16.5 | 7.97 | 103.1 | 17.6 | 8.00 | 103.1 | 16.6 | 7.99 | 101.6 | 17.4 | 8.03 | 105.4 | 15.9 | 7.97 | 102.1 | 16.2 | 8.06 | 106.1 | 16.3 | 8.04 | 103.5 |
| B4 | 15.8 | 7.99 | 101.3 | 17.9 | 8.00 | 102.9 | 16.3 | 7.99 | 103.4 | 17.2 | 8.04 | 107.2 | 15.8 | 7.97 | 104.3 | 16.3 | 8.07 | 108.7 | 16.1 | 8.04 | 103.4 |
| Wb B | 16.3 | 7.97 | 99.9 | 18.1 | 8.00 | 101.7 | 16.6 | 7.99 | 102 | 17.6 | 8.02 | 104.3 | 16 | 7.98 | 103.4 | 16.7 | 8.08 | 112.4 | 16.3 | 8.04 | 105.7 |
| C1 | 16.8 | 7.96 | 102.9 | 17.4 | 8.01 | 101.2 | 16.5 | 8.00 | 101.8 | 20.3 | 7.96 | 106.3 | 16 | 7.96 | 101.7 | 16.2 | 8.05 | 108 | 16.5 | 8.05 | 106.4 |
| C2 | 16.1 | 7.98 | 102.5 | 17.9 | 8.00 | 102.9 | 16.4 | 8.00 | 103.3 | 19.5 | 7.98 | 106.4 | 15.9 | 7.96 | 102.1 | 16.2 | 8.05 | 108.1 | 16.6 | 8.04 | 103.2 |
| C3 | 16.5 | 7.97 | 102.6 | 17.2 | 8.02 | 100.3 | 16.2 | 8.01 | 101.3 | 19.2 | 7.97 | 105.1 | 16 | 7.96 | 101.3 | 16.1 | 8.05 | 107 | 16.5 | 8.03 | 104.8 |
| C4 | 16.2 | 7.98 | 101.4 | 18 | 8.00 | 101.2 | 16.5 | 8.00 | 102.5 | 19.4 | 7.97 | 105.8 | 16 | 7.96 | 102.2 | 16.2 | 8.04 | 105.9 | 16.2 | 8.03 | 102.6 |
| Wb C | 16.5 | 7.97 | 102.1 | 18 | 8.00 | 101 | 16.6 | 8.00 | 101.8 | 22.2 | 7.94 | 107.8 | 16.1 | 7.97 | 102.5 | 16.4 | 8.07 | 110.9 | 16.4 | 8.04 | 105.8 |
| D1 | 16.5 | 7.98 | 102.2 | 17.4 | 8.02 | 100.7 | 16.5 | 8.00 | 101.9 | 23.7 | 7.90 | 107.9 | 16.2 | 7.96 | 101 | 16.6 | 8.03 | 106.4 | 16.7 | 8.02 | 103.4 |
| D2 | 16 | 7.99 | 101.4 | 17.9 | 8.00 | 102.3 | 16.6 | 8.00 | 101.5 | 23.3 | 7.89 | 108.2 | 16 | 7.95 | 101.1 | 16.2 | 8.04 | 105.9 | 16.6 | 8.02 | 102.3 |
| D3 | 16.4 | 7.98 | 103.2 | 17.4 | 8.01 | 101 | 16.4 | 8.00 | 102.3 | 23.4 | 7.90 | 106.8 | 16 | 7.96 | 100.5 | 16.5 | 8.05 | 105.5 | 16.8 | 8.03 | 104.9 |
| D4 | 16 | 7.98 | 102.3 | 17.9 | 8.01 | 102.8 | 16.6 | 8.00 | 103.5 | 23.5 | 7.90 | 107.5 | 16 | 7.96 | 101.6 | 16.2 | 8.05 | 108.2 | 16.5 | 8.04 | 105.1 |
| Wb D | 16.5 | 7.97 | 101.9 | 18 | 8.00 | 101.7 | 17.4 | 7.99 | 103.4 | 29.5 | 7.82 | 110.5 | 16.2 | 7.96 | 101.7 | 16.5 | 8.07 | 109.5 | 17.1 | 8.03 | 107.6 |
| E1 | 16.5 | 7.97 | 102.5 | 16.6 | 8.03 | 99.8 | 16.4 | 8.00 | 101.7 | 20 | 7.89 | 101.5 | 16.7 | 7.95 | 102 | 16.3 | 8.04 | 105.6 | 16.6 | 8.02 | 102.7 |
| E2 | 15.6 | 7.99 | 101.2 | 16.5 | 8.04 | 99.2 | 16.2 | 8.01 | 101.2 | 18.1 | 7.94 | 102.3 | 15.9 | 7.96 | 101.4 | 16.5 | 8.04 | 105.9 | 16.6 | 8.02 | 102.9 |
| E3 | 16.2 | 7.97 | 102.7 | 16.5 | 8.03 | 99.8 | 16.3 | 8.00 | 102.1 | 20.3 | 7.98 | 107.6 | 16.4 | 7.96 | 102.1 | 16.2 | 8.04 | 105.6 | 16.5 | 8.03 | 104.6 |
| E4 | 15.4 | 7.99 | 101.3 | 16.5 | 8.03 | 100 | 16.2 | 8.01 | 101.5 | 19 | 8.00 | 106.9 | 16.1 | 7.98 | 101.9 | 16.6 | 8.04 | 105.6 | 16.6 | 8.02 | 103.4 |
| Wb E | 16 | 7.98 | 101.6 | 16.7 | 8.03 | 100.2 | 16.3 | 8.00 | 100.8 | 21.5 | 7.96 | 107.4 | 16.3 | 7.97 | 102.5 | 16.4 | 8.04 | 106.4 | 16.5 | 8.04 | 104.6 |
| F1 | 16.4 | 7.97 | 103.5 | 16.7 | 8.03 | 101 | 16.6 | 8.00 | 102.9 | 23.6 | 7.90 | 109.1 | 16.6 | 7.95 | 101.7 | 16.9 | 8.06 | 106.2 | 16.5 | 8.03 | 104.1 |
| F2 | 15.6 | 7.99 | 102.3 | 16.5 | 8.04 | 100.6 | 16.4 | 8.01 | 102 | 19.6 | 7.96 | 106.8 | 15.9 | 7.96 | 100.8 | 16.7 | 8.04 | 105.2 | 16.5 | 8.04 | 104.3 |
| F3 | 16.1 | 7.98 | 101.9 | 16.5 | 8.04 | 99.7 | 16.4 | 8.00 | 101.9 | 23.3 | 7.91 | 108 | 16.4 | 7.95 | 100.5 | 16.5 | 8.04 | 104.9 | 16.4 | 8.02 | 99.6 |
| F4 | 15.5 | 7.99 | 101.1 | 16.4 | 8.04 | 99.2 | 16.4 | 8.00 | 101.7 | 22.7 | 7.88 | 108.3 | 16 | 7.95 | 99.5 | 16.7 | 8.03 | 104.8 | 16.5 | 8.02 | 101.8 |
| Wb F | 16.1 | 7.98 | 101.9 | 16.8 | 8.03 | 103 | 16.3 | 8.00 | 101.1 | 30.1 | 7.82 | 113.3 | 16.2 | 7.96 | 100.7 | 16.8 | 8.05 | 108.9 | 16.4 | 8.03 | 102.9 |
| G1 | 16 | 7.98 | 101.9 | 16.7 | 8.03 | 99.9 | 16.6 | 8.00 | 101.2 | 21.2 | 7.94 | 109.2 | 16.4 | 7.95 | 100.6 | 16.7 | 8.03 | 106.6 | 16.7 | 8.02 | 102.6 |
| G2 | 16.1 | 7.98 | 101.7 | 16.7 | 8.03 | 99.4 | 16.6 | 8.00 | 101 | 22 | 7.93 | 108.7 | 16.6 | 7.96 | 102.6 | 16.2 | 8.05 | 104.9 | 16.3 | 8.02 | 102.6 |
| G3 | 15.9 | 7.98 | 102.3 | 16.6 | 8.03 | 100 | 16.5 | 8.00 | 101.4 | 21.2 | 7.97 | 109.6 | 16.3 | 7.96 | 101.9 | 16.6 | 8.05 | 106.6 | 16.5 | 8.05 | 106.4 |
| G4 | 16.1 | 7.98 | 103.4 | 16.7 | 8.03 | 101.2 | 16.5 | 8.00 | 101.8 | 21.7 | 7.95 | 110.3 | 16.5 | 7.97 | 101.8 | 16.2 | 8.05 | 106.2 | 16.1 | 8.05 | 105.3 |
| Wb G | 16.2 | 7.98 | 102.4 | 16.9 | 8.03 | 100.8 | 16.5 | 8.00 | 101.2 | 26.1 | 7.88 | 113.3 | 16.5 | 7.96 | 101.6 | 16.5 | 8.06 | 108.8 | 16.5 | 8.04 | 106.1 |
| H1 | 16.3 | 7.97 | 103.6 | 17.2 | 8.02 | 104.7 | 16.7 | 8.00 | 102.9 | 18 | 8.03 | 109.6 | 16.6 | 7.97 | 103.2 | 17 | 8.06 | 110.1 | 16.5 | 8.04 | 104.8 |
| H2 | 16 | 7.98 | 102.9 | 16.8 | 8.04 | 101.2 | 16.6 | 8.01 | 101.5 | 18.4 | 8.03 | 105.3 | 16.7 | 7.97 | 103.2 | 16.2 | 8.06 | 106.7 | 16.3 | 8.04 | 104 |
| H3 | 16.2 | 7.98 | 102.5 | 17 | 8.03 | 101 | 16.6 | 8.00 | 101.2 | 17.9 | 8.03 | 105.5 | 16.4 | 7.96 | 102.3 | 16.9 | 8.05 | 105.7 | 16.6 | 8.07 | 109.8 |
| H4 | 16.1 | 7.98 | 101.7 | 16.8 | 8.03 | 99 | 16.6 | 8.00 | 101.4 | 18.4 | 7.99 | 101.8 | 16.6 | 7.94 | 102 | 16 | 8.04 | 104 | 16.2 | 8.04 | 105.1 |
| Wb H | 16.4 | 7.97 | 101.8 | 17.3 | 8.02 | 100.6 | 16.6 | 8.00 | 101.4 | 18.3 | 8.01 | 104.9 | 16.6 | 7.96 | 102.8 | 16.5 | 8.07 | 109.1 | 16.5 | 8.05 | 106.4 |
| Header A | 17.1 | 7.96 | 103.2 | 17.6 | 8.01 | 102.5 | 16.3 | 8.00 | 101.6 | 16.5 | 8.01 | 98.5 | 15.3 | 7.95 | 101.2 | 15.4 | 8.05 | 102.3 | 15.9 | 8.02 | 99.3 |
| Header B | 16.7 | 7.97 | 103.7 | 16.8 | 8.03 | 99.3 | 16.6 | 7.99 | 102 | 16.8 | 8.01 | 100.1 | 15.6 | 7.95 | 99.9 | 15 | 8.06 | 103.6 | 15.4 | 8.03 | 97.8 |
| Header C | 16.8 | 7.97 | 103.7 | 17.6 | 8.01 | 102.3 | 16.8 | 8.00 | 102.9 | 16.6 | 8.01 | 99.8 | 15.2 | 7.95 | 100.8 | 15.6 | 8.05 | 103.8 | 15.6 | 8.02 | 99.4 |
| Header D | 16.7 | 7.96 | 104.2 | 16.6 | 8.02 | 99.1 | 16.6 | 7.99 | 101.7 | 17 | 8.00 | 99.3 | 15.6 | 7.95 | 99 | 14.8 | 8.05 | 103.6 | 15.1 | 8.03 | 97.8 |
| Header E | 16.9 | 7.95 | 104.4 | 16 | 8.04 | 97.9 | 15.5 | 8.02 | 98.6 | 15.2 | 8.01 | 97 | 15.4 | 7.96 | 99.6 | 16.6 | 8.04 | 105 | 15.8 | 8.02 | 99.2 |
| Header F | 16.5 | 7.97 | 103.6 | 16.2 | 8.03 | 97.8 | 15.2 | 8.02 | 98.6 | 18.5 | 7.99 | 102.7 | 16.6 | 7.94 | 101.5 | 16 | 8.04 | 103.8 | 16.1 | 8.02 | 100.6 |
| Header G | 16.3 | 7.97 | 102.7 | 16.3 | 8.03 | 98.2 | 15.8 | 8.01 | 100 | 18.4 | 7.99 | 102.2 | 16.5 | 7.94 | 101.8 | 15.7 | 8.04 | 103.5 | 16.2 | 8.02 | 100.3 |
| Header H | 16.6 | 7.96 | 102.7 | 16.3 | 8.03 | 98.5 | 15.8 | 8.01 | 99.7 | 16.5 | 8.01 | 99.3 | 15.8 | 7.95 | 100.1 | 16.6 | 8.03 | 105.2 | 15.5 | 8.03 | 99.3 |
