# Supplementary material for: Light limitation and water velocity modify the impacts of simulated marine heatwaves on juvenile giant kelp
Source: J Phycol. 2025 Jul 18;61(5):1173–94. doi: 10.1111/jpy.70054 (PMC12547647; doi:10.1111/jpy.70054)
Supplement: Supplementary file 12 — Table S6. Ammonium (NH4 +), total NOx, nitrite (NO2 −), and nitrate (NO3 −) concentrations (μg · L−1) of water samples taken from the experimental tanks and headers throughout the experimental period. [file JPY-61-1173-s005.docx]

| Date | 14/02/2023 | | | | 28/02/2023 | | | | 21/03/2023 | | | | 11/04/2023 | | | |
| --- | --- | --- | --- | --- | --- | --- | --- | --- | --- | --- | --- | --- | --- | --- | --- | --- |
| Tank | NH_4_^+^ (µg L^-1^) | NO_x_  (µg L^-1^) | NO_2_^−^  (µg L^-1^) | NO_3_^−^  (µg L^-1^) | NH_4_^+^  (µg L^-1^) | NO_x_  (µg L^-1^) | NO_2_^−^  (µg L^-1^) | NO_3_^−^  (µg L^-1^) | NH_4_^+^  (µg L^-1^) | NO_x_  (µg L^-1^) | NO_2_^−^  (µg L^-1^) | NO_3_^−^  (µg L^-1^) | NH_4_^+^  (µg L^-1^) | NO_x_  (µg L^-1^) | NO_2_^−^  (µg L^-1^) | NO_3_^−^  (µg L^-1^) |
| A1 | 6.46 | 72.36 | 4.32 | 68.04 | 13.17 | 41.69 | 3.91 | 37.78 | 8.07 | 130.04 | 6.12 | 123.92 | 2.93 | 84.89 | 5.06 | 79.83 |
| B1 | 6.32 | 73.23 | 4.90 | 68.33 | 6.20 | 109.55 | 4.20 | 105.35 | 7.09 | 106.47 | 4.84 | 101.63 | 9.64 | 93.25 | 5.78 | 87.47 |
| C1 | 5.23 | 65.49 | 3.55 | 61.94 | 6.16 | 128.59 | 5.92 | 122.67 | 5.23 | 89.82 | 5.74 | 84.08 | 9.73 | 82.43 | 6.07 | 76.37 |
| D1 | 22.25 | 76.47 | 4.66 | 71.80 | 6.81 | 115.11 | 4.85 | 110.27 | 4.66 | 85.27 | 3.99 | 81.28 | 19.53 | 127.37 | 5.55 | 121.82 |
| E1 | 4.31 | 67.02 | 3.60 | 63.41 | 5.13 | 116.52 | 5.59 | 110.93 | 4.73 | 100.37 | 5.86 | 94.51 | 7.16 | 69.34 | 5.27 | 64.07 |
| F1 | 4.57 | 67.93 | 4.09 | 63.84 | 4.13 | 89.88 | 3.31 | 86.57 | 3.68 | 109.77 | 5.60 | 104.16 | 8.07 | 96.04 | 6.09 | 89.95 |
| G1 | 5.42 | 75.67 | 7.27 | 68.40 | 7.41 | 115.12 | 5.80 | 109.32 | 8.02 | 105.11 | 5.83 | 99.28 | 5.56 | 61.09 | 3.97 | 57.12 |
| H1 | 3.55 | 73.64 | 4.59 | 69.05 | 7.23 | 111.96 | 5.43 | 106.53 | 2.74 | 106.45 | 5.23 | 101.22 | 3.88 | 70.36 | 4.35 | 66.00 |
| Header A |  |  |  |  | 5.51 | 117.18 | 4.53 | 112.65 | 9.26 | 111.59 | 5.38 | 106.21 | 7.66 | 78.27 | 5.39 | 72.88 |
| Header B |  |  |  |  | 11.02 | 112.25 | 4.83 | 107.41 | 3.84 | 86.52 | 3.74 | 82.78 | 5.30 | 96.68 | 6.96 | 89.72 |
| Header C |  |  |  |  | 6.44 | 118.29 | 5.30 | 112.98 | 28.05 | 119.64 | 5.25 | 114.40 | 6.33 | 81.19 | 5.09 | 76.11 |
| Header D |  |  |  |  | 8.92 | 116.61 | 4.95 | 111.66 | 3.84 | 105.43 | 5.25 | 100.18 | 7.03 | 77.87 | 4.74 | 73.13 |
| Header E |  |  |  |  | 4.30 | 134.95 | 5.69 | 129.27 | 4.40 | 91.60 | 4.81 | 86.79 | 5.61 | 100.79 | 7.17 | 93.62 |
| Header F |  |  |  |  | 3.66 | 112.04 | 4.32 | 107.72 | 4.93 | 96.42 | 4.86 | 91.56 |  |  |  |  |
| Header G |  |  |  |  | 10.77 | 112.44 | 5.26 | 107.18 | 4.73 | 90.59 | 5.31 | 85.28 | 6.97 | 97.26 | 6.35 | 90.90 |
| Header H |  |  |  |  | 10.61 | 115.75 | 4.95 | 110.79 | 4.98 | 98.92 | 3.84 | 95.08 |  |  |  |  |
